# A STING agonist prodrug reprograms tumor-associated macrophage to boost colorectal cancer immunotherapy

**DOI:** 10.7150/thno.101001

**Published:** 2025-01-01

**Authors:** Aohua Deng, Renming Fan, Yongrui Hai, Junyan Zhuang, Bingjie Zhang, Xintong Lu, Wenhui Wang, Li Luo, Ge Bai, Lei Liang, Le Yang, Minggao Zhao, Gaofei Wei

**Affiliations:** 1 Institute of Medical Research, Northwestern Polytechnical University, Xi'an, 710072, China.; 2 Research & Development Institute of Northwestern Polytechnical University, Shenzhen, 518057, China.; 3 Institute of Biopharmaceutical and Health Engineering, Shenzhen International Graduate School, Tsinghua University, Shenzhen, 518055, China.; 4 Precision Pharmacy & Drug Development Center, Department of Pharmacy, Tangdu Hospital, Air Force Military Medical University, Xi'an, 710038, China.; 5 Department of Colorectal Surgery, Fudan University Shanghai Cancer Center, Shanghai, 200032, China.

**Keywords:** prodrug, STING agonist, TREM2 inhibitor, tumor associated macrophage, colorectal cancer

## Abstract

**Rationale:** Tumor-associated macrophages (TAMs) are abundant in colorectal cancer (CRC), correlating with immunosuppression and disease progression. Activation of the stimulator of interferon gene (STING) signaling pathway in TAMs offers a promising approach for CRC therapy. However, current STING agonists face challenges related to tumor specificity and administration routes.

**Method:** The Cancer Genome Atlas (TCGA) database analysis and multicolor immunofluorescence experiments of human CRC samples analysed triggering receptor expressed on myeloid cells 2 (TREM2) expression in the tumor microenvironment of CRC patients. We designed and synthesized a STING agonist prodrug GB2 to reprogram TAMs by targeting TREM2 in tumors. Preliminary evaluation of the anti-tumor capacity of prodrug GB2 in the mouse CRC model intravenously. RNA-seq analysis of bone marrow-derived macrophages (BMDM) after GB2 treatment reveals novel pharmacological mechanisms for the prodrug GB2.

**Results:** Over-expressed TREM2 in TAMs correlates with CRC progression. Via targeting TREM2 expressed in TAMs, GB2 induces comprehensive tumor regression by administrating intravenously in mouse colon cancer models, as well as in a STING^low^ mouse melanoma model, with no systemic toxicity. Upon treatment with GB2, TAMs exhibit an M1 phenotype with pro-inflammatory function and demonstrate enhanced phagocytosis capacity. The molecular mechanisms involve (1) GB2 upregulating the Glycolysis-ROS-HIF-1α axis, thereby promoting glucose metabolism and inflammatory cytokine expression; (2) GB2 inducing endoplasmic reticulum-mitochondria contact (MERC), leading to mitochondrial fission, ultimately facilitating Ca^2+^-mediated phagocytosis. Besides, GB2-treated macrophages reverse immunosuppression, facilitating CD8^+^ T cell tumor infiltration and effector function. Combining GB2 with αPD-1 therapy reveals a synergistic effect on tumor inhibition, leading to prolonged mouse survival.

**Conclusion:** By targeting TREM2 and activating the STING signaling pathway in TAMs, prodrug GB2 exhibits excellent anti-tumor efficacy and immune-activating capacity in the mouse colon cancer model.

## Introduction

The immune system offers robust defense against tumor progression, and immunotherapy has the transformative potential to reshape the tumor immune landscape 1-5. Within colorectal tumors, TAMs are particularly enriched, and their intrinsic characteristics play pivotal roles in determining the fate of tumors 6, 7. Immunostimulatory macrophages, usually referred to as M1-like macrophages, present a pro-inflammatory and anti-tumor action mode; while their counterparts, M2-like macrophages, are immunosuppressive and generally contribute to tumor progression 8-10. Given the high plasticity of tumor-associated macrophages (TAMs), strategic utilization of immune agents can manipulate macrophage phenotypes effectively, orchestrate the immune landscape and boost immunotherapy in colorectal cancer (CRC) 6-8.

Recent studies have focused on reprogramming TAMs by activating the stimulator of interferon gene (STING), which elicits innate immunity to curb tumor development 11. Mechanistically, STING activation initiates signaling transduction primarily through interaction with the TBK1 kinase, subsequently involving transcription factors IRF3, resulting in the upregulated production of type I interferon (IFN-Ⅰ) and other proinflammatory cytokines 11, 12. STING-activation reverses TAMs from M2-like to M1-like phenotype, which holds a large potential for adaptative anti-tumor immune response in CRC.

Numerous natural and synthetic STING agonists are developed as effective immune-activating drugs, and stepping into clinical research 13, 14. Natural STING agonists, mainly cyclic dinucleotides (CDN), exhibit potent STING activation but remain challenging in structural stability and quick clearance following administration 15. In contrast, synthetic STING agonists, exemplified by MSA-2, are non-CDN drugs that activate STING via triggering conformational change, which displays noteworthy efficacy in tumor inhibition and immune response 16-18. Despite the promise, STING agonist MSA-2 faces challenges. Non-targeting activation and systemic cytokine storms have been reported as side effects, due to the widespread expression of STING in both tumor and normal tissues 19, 20. Hence, targeting activation of synthetic STING agonists in tumors is vital for improving drug efficacy and ameliorating side effects. Additionally, MSA-2 encounters limitations in oral bioavailability and cytosolic entry, potentially compromising its antitumor efficacy 16, 21. This underscores the urgent need for developing a more efficient and tumor-specific STING agonist capable of effectively regulating TAMs.

Recent studies have identified TREM2 as a receptor expressed on TAMs contributing to immunosuppression and malignant progression 22, 23. TREM2 loss of function, via gene depletion or neutralized antibody, alters the macrophage landscape within tumors and enhances the efficacy of immune checkpoint blockade 22-24. Therefore, we wondered whether TREM2-expressing TAMs can be leveraged to enhance the efficacy of MSA-2 on immunotherapy while minimizing systemic toxic side effects.

Based on the above hypothesis, this study introduces a novel immunomodulating agent named GB2, utilizing a TREM2 inhibitor that links MSA-2 with a disulfide bond. High glutathione (GSH) in the tumor microenvironment reacts with disulfide bonds, making GB2 achieve a tumor-specific response. GB2 allows for intravenous administration, ensuring tumor-responsive STING activation and guaranteeing both the safety and efficacy of the drug. We disclose that synergistic effect on TREM2 inhibition and STING activation elicits innate and adaptive anti-tumor immunity by reprogramming TAMs. Notably, prodrug GB2 enhances the therapeutic responsiveness to αPD-1 treatment, leading to tumor eradication and survival prolongation in the murine colon cancer MC38 model. This study proposes a novel therapeutic strategy by targeting TREM2-expressing TAMs and activating STING to optimize tumor immunity.

## Results

### TREM2 is highly expressed in macrophages and correlates with poor prognosis in CRC patients

TREM2, a myeloid receptor transmitting intracellular signals that sustain microglial responses during Alzheimer's disease, has recently gained attention for its association with tumor progression and immune response 24-26. It has been reported that overexpressed TREM2 in breast cancer and colon cancer linking to poor prognosis 23. Trem2^-/-^ mice demonstrate enhanced resistance to tumor growth in models of 3-methylcholanthrene-induced sarcoma, colorectal cancer, and mammary tumors 23, 27, 28. Using anti-TREM2 monoclonal antibodies drives robust anti-tumor immunity by increasing CD8^+^ tumor-infiltrating lymphocytes (TILs) infiltration and enhancing their effector function in glioblastoma ovarian cancer 22, 23.

**Figure 1:**
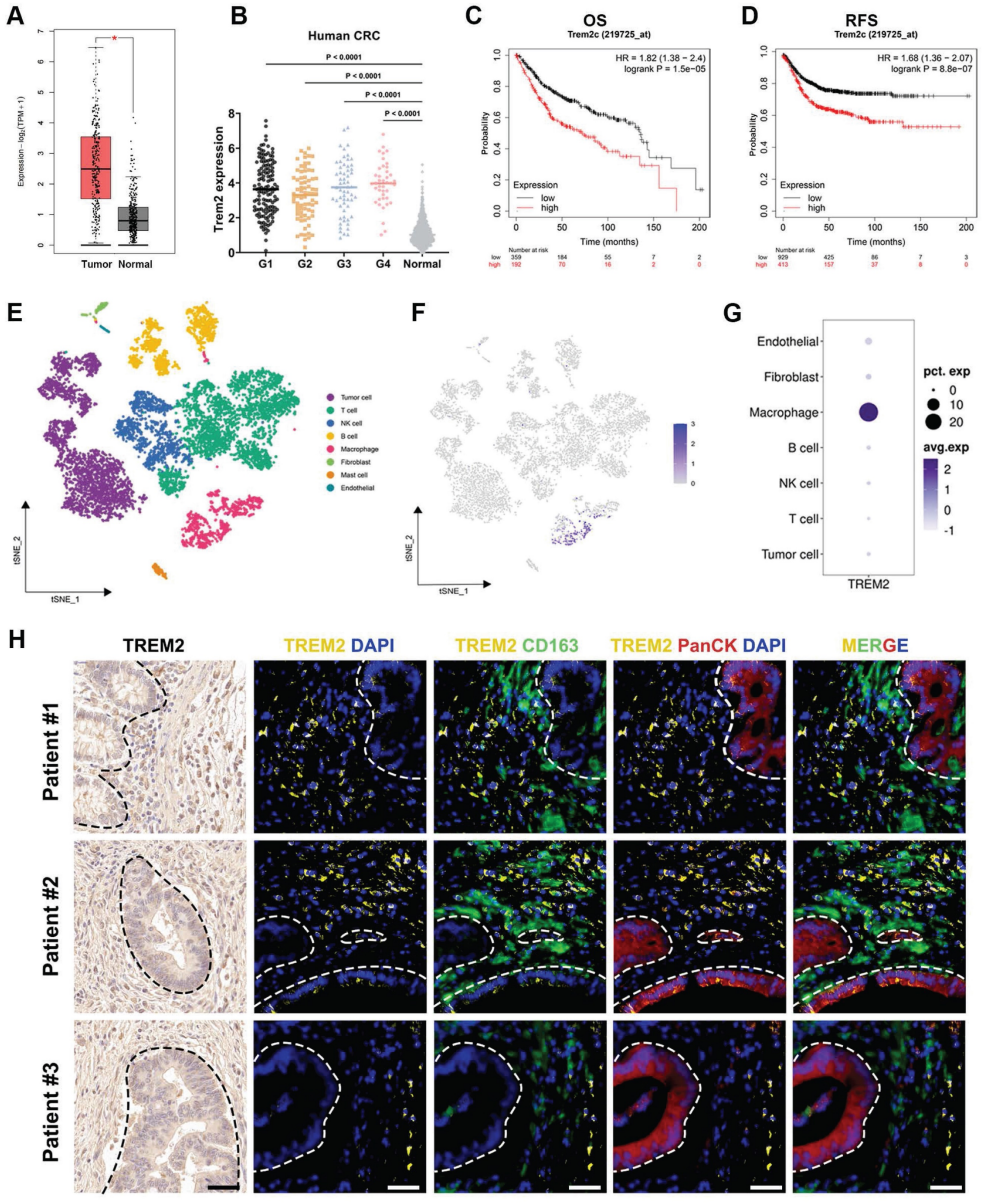
TREM2 is highly expressed in macrophages and correlates with poor prognosis in CRC patients. (A) Box plots illustrating TREM2 expression in colon adenocarcinoma tumors (*n* = 275) compared to normal specimens (n = 349). (B) TCGA database analysis depicting TREM2 expression in human CRC with different grades: G1 (*n* = 136), G2 (*n* = 74), G3 (*n* = 59), G4 (*n* = 41), and normal tissue (*n* = 779). (C and D) Kaplan-Meier curves presenting an OS (n = 1061) and RFS (n = 1336) of colon cancer patients based on TREM2 expression. (E) Plots revealing cell clusters identified in human CRC tumors by t-SNE analysis. (F) Dot plots displaying TREM2 expression level in each cluster. (G) TREM2 expression level in each cluster by *t*-SNE analysis. (H) Multiple immunofluorescence (IF) staining for TREM2 expression in three human CRC samples. CD163 represents human macrophages. PanCK denotes tumor cells. DAPI indicates nuclei. White dash lines indicate the boundary of CRC tumors and TAMs. Scale bars: 50 μm.

We conducted a comprehensive analysis utilizing the gene expression profiling interactive analysis (GEPIA) database to elucidate the clinical significance of TREM2 expression in human CRC. The results underscore a significant elevation in TREM2 gene level within tumor tissues compared to normal counterparts (Figure 1A). Likewise, TREM2 is also in higher expression in tumors across various pathological grading than that in normal tissue, according to the cancer genome atlas (TCGA) database (Figure 1B). Examining the correlation between elevated TREM2 expression and clinical outcomes, we stratified colon cancer cases into TREM2^high^ and TREM2^low^ groups and plotted prognosis curves in Kaplan-Meier Plotter. Results reveal that high TREM2 expression correlates with shorter median refractory free survival (RFS) and overall survival (OS) in colon cancer (Figure 1C-D).

To identify the specific cell subtypes expressing TREM2 in CRC tumors, we examine the single-cell RNA sequencing (scRNA-seq) dataset using the Seurat R package based on published data. Employing t-distributed stochastic neighbor embedding (t-SNE) facilitated the identification of distinct cell clusters, revealing seven cell types beyond the tumor context, including T cells, B cells, macrophages, natural killer (NK) cells, fibroblasts, mast cells, and endothelial cells (Figure 1E). Notably, within these cell clusters, TREM2 shows specifically high expression in macrophages, rather than tumor cells (Figure 1F-G).

Three human CRC specimens were selected to validate such discovery by assessing the co-expression of TREM2 and macrophage marker CD163 in tumor tissue. IF staining results display that macrophages are quite abundant in tumor tissue, with all three patient specimens displaying positive staining for TREM2. Across these specimens, TREM2 predominantly localized to areas with CD163^+^ macrophages rather than cytokeratin (PanCK)-positive tumor cells (Figure 1H). Previous reports have revealed that TREM2-expressing macrophages adopt an M2-like phenotype and positively correlate with tumor progression 29. Hence, targeting TREM2 in tumor-associated macrophages might be a feasible therapy approach.

### Design and characterization of GB2

**Figure 2:**
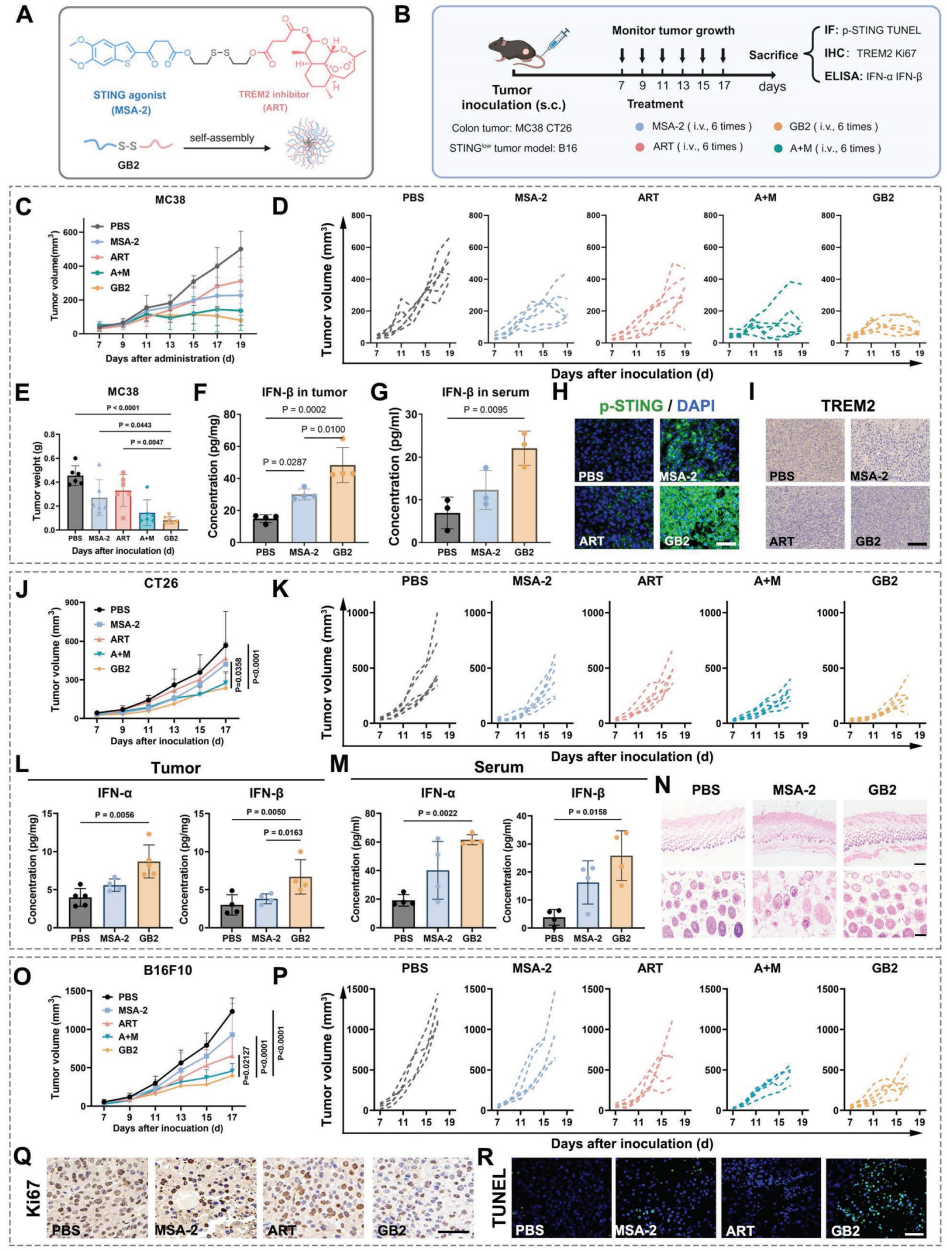
Pharmacological analysis for GB2 in mouse tumor models. (A) Structure of GB2, where the blue part represents the STING agonist MSA-2, and the red part represents TREM2 inhibitor ART. (B) Experimental design: C57BL/6 mice were inoculated with MC38 cells and treated with PBS, MSA-2 (4 mg/kg, e.q.), ART (6 mg/kg, e.q.), GB2 (10 mg/kg) and combination of MSA-2 (4 mg/kg) and ART (6 mg/kg) (A+M). BALB/C mice were inoculated with CT26 cells and treated with PBS, MSA-2 (4 mg/kg, e.q.), and GB2 (10 mg/kg). C57BL/6 mice were inoculated with B16F10 cells and treated with PBS, MSA-2 (4 mg/kg, e.q.), ART (6 mg/kg, e.q.), and GB2 (10 mg/kg). Administration for mice bearing tumors began when tumor size reached about 50 mm^3^. (C) Growth curve for MC38 tumors with indicated treatment (n = 6). (D) Growth of individual MC38 tumors (n = 6). (E) Weight of MC38 tumors treated with PBS, MSA-2, ART, A+M, and GB2 at day 17 (n = 6). (F) Concentration of IFN-β in tumor from mice with PBS, MSA-2, and GB2 (n = 4). (G) Concentration of IFN-β in serum from mice (n = 3). (H) IF staining for MC38 tumors, staining with *p*-STING and DAPI. Scale bar, 50 μm. (I) IF staining for tumors, staining with *p*-STING and DAPI. Scale bar, 100 μm. (J) Growth curve for CT26 tumor with indicated treatment (n = 6). (K) Growth of individual CT26 tumors (n = 6). (L-M) Concentration of IFN-α and IFN-β in tumor (L) and serum (M) from mice (n = 3). (N) H&E staining for skin from mice treated with PBS, MSA-2 (10 mg/kg), and GB2 (10 mg/kg). Scale bars: 500 μm (4×), 200 μm (10×). (O) Growth curve for B16F10 tumors (n = 6). (P) Growth of individual B16F10 tumors (n = 6). (Q) IHC staining for proliferation index Ki67 in B16F10 tumors. Scale bar: 50 μm (R) IF staining for apoptosis marker TUNEL in B16F10 tumors. Scale bars: 100 μm. n indicates biological replicates. Error bars represent means ± SD. Differences between groups were tested using one-way ANOVA followed by Tukey's multiple comparisons test.

Considering that TREM2-expressing TAMs are abundant in CRC and associated with poor prognosis and immune suppression, we hypothesized that targeting TREM2 in TAMs could augment therapeutic effect of STING agonist MSA-2 while minimizing systemic toxic side effects. Consequently, we have developed a novel immunomodulating agent named GB2, which links MSA-2 with a newly characterized TREM2 inhibitor, artesunate (ART) 25. The synthetic routes were listed in Scheme S1. Via a GSH responsive disulfide bond, GB2 is able to be self-assembled (Figure 2A). The compound structure of GB2 and synthetic intermediate GB1 was confirmed by nuclear magnetic resonance (NMR) and high-resolution mass spectrometry (HRMS) (Figure S1-S5).

When dispersed in PBS, GB2 presented better solubility than positive drug MSA-2, and a Tyndall effect was observed, indicating GB2 formed a colloidal solution (Figure S6A). We deduced that GB2 might be self-assembled in PBS solution and utilized transmission electron microscopy (TEM) to identify the solution. GB2 displayed a spherical structure, certifying that GB2 aggregated together in the dispersing system (Figure S6B-C). When determining particle size utilizing a dynamic light scattering system, GB2 demonstrated a uniform particle size at 151.6 nm and a minor polydispersity index (PDI) at 0.17 (Figure S6D). Charge distribution experiments disclosed that GB2 maintained a negative potential at -36.42 mV, as Figure S6E depicted**,** indicating a stable dispersal. GB2 solution maintains fine colloidal stability unless GSH is added (Figure S6A). The spherical structure completely collapses when mixed with GSH, confirming its GSH-responsive nature (Figure S6C). We next determined drug release via high performance liquid chromatography (HPLC). Results showed that GB2 remained stable for 24 h in a biological environment and released drugs once supplemented with GSH (Figure S6F-G).

The above results proved that GB2 is endowed with metabolic stability in drug circulation and provided the feasibility of intravenous administration in mice.

### Intravenous injection of GB2 induces tumor rejection in murine CRC and melanoma models

To verify the tumor-specific effect of GB2, we conducted biodistribution experiments using a near-infrared fluorescent dye for *in vivo* imaging. As is shown in Figure S7A, GB2 is enriched in tumor area in 3 h after intravenous administration and lasted for 24 h. Overall, we conducted subsequent pharmacological investigations of prodrug GB2 via employing two murine models of colon cancer (MC38 and CT26) and a STING^low^ murine melanoma model B16F10. The administration schedule is outlined in Figure 2B.

In the MC38 model, prodrug GB2 demonstrated a significant improvement in tumor regression compared to a single treatment of MSA-2 or ART. Notably, such results were consistent with the combinational treatment of MSA-2 and ART (group A+M) at an equivalent dose (Figure 2C). The growth curves for the respective groups are depicted in Figure 2D, and tumor weight analysis revealed that tumors in the GB2 group had the lowest weight (Figure 2E). Hematoxylin and eosin (H&E staining on tumor slices proves the biosafety of GB2 administration (Figure S7B). Furthermore, the evaluation of IFN-β content in tumor tissues and blood serum revealed a substantial increase in GB2 group (Figure 2F-G). The upregulation of *p*-STING expression in tumor tissues indicated the activation of the STING signaling pathway (Figure 2H). Additionally, immunohistochemistry (IHC) analysis demonstrated a noticeable downregulation of TREM2 expression in the GB2 group (Figure 2I).

Similar evaluations were performed in the CT26 model, yielding consistent results. Prodrug GB2 exhibited superior tumor inhibition compared to MSA-2 treatment at an equivalent dose, with negligible impact on mouse body weight (Figure 2J-K and Figure S7C-D). Determination of IFN-Ⅰ content in tumor and serum revealed a higher IFN-I level in the prodrug GB2 group (Figure 2L-M). Except, skin inflammation is found in MSA-2 group while treated subcutaneously in the same dose. H&E staining reveals that MSA-2 induced local fibrosis and epidermal layer thickening in the skin, probably due to non-targeting activation of STING in the whole organism (Figure 2N). While in GB2 group, little systematic damage is found in the skin because GB2 is a highly tumor-responsive prodrug.

Given that melanoma is considered an intrinsic STING signaling impairment solid tumor, potentially inhibited by DNA methylation, conventional STING agonists have limited therapeutic effects on it 30, 31. Considering GB2's bi-functional nature, its efficacy was evaluated in a mouse melanoma model by subcutaneously implanting B16F10 cells in C57 mice. Growth curve recordings and tumor weight measurements demonstrated the extraordinary effect of prodrug GB2 on tumor restriction (Figure 2O-P and Figure S7E). IHC staining for tumor proliferation marker Ki67 as well as immunofluorescence (IF) staining for damage marker TUNEL revealed that GB2 delays tumor growth and induces significant tumor damage (Figure 2Q-R). To explore the potential for clinical translation, we conducted pharmacological experiments on human-derived colon tumor cell lines HCT116. As Figure S7F-H showed, prodrug GB2 significantly inhibited tumor growth in HCT11nude mice and performed better than any single dose or combinational administration.

Above all, our findings indicate that intravenous injection of GB2 raises a strong tumor regression in both colorectal cancer and STING^low^ melanoma models. These have proven that utilizing TREM2 inhibitor ART indeed augments the therapeutic outcome of STING agonist MSA-2, and the prodrug GB2 is a promising anti-tumor agent.

### Transcriptome analysis identifies the key modulator role of macrophages

**Figure 3:**
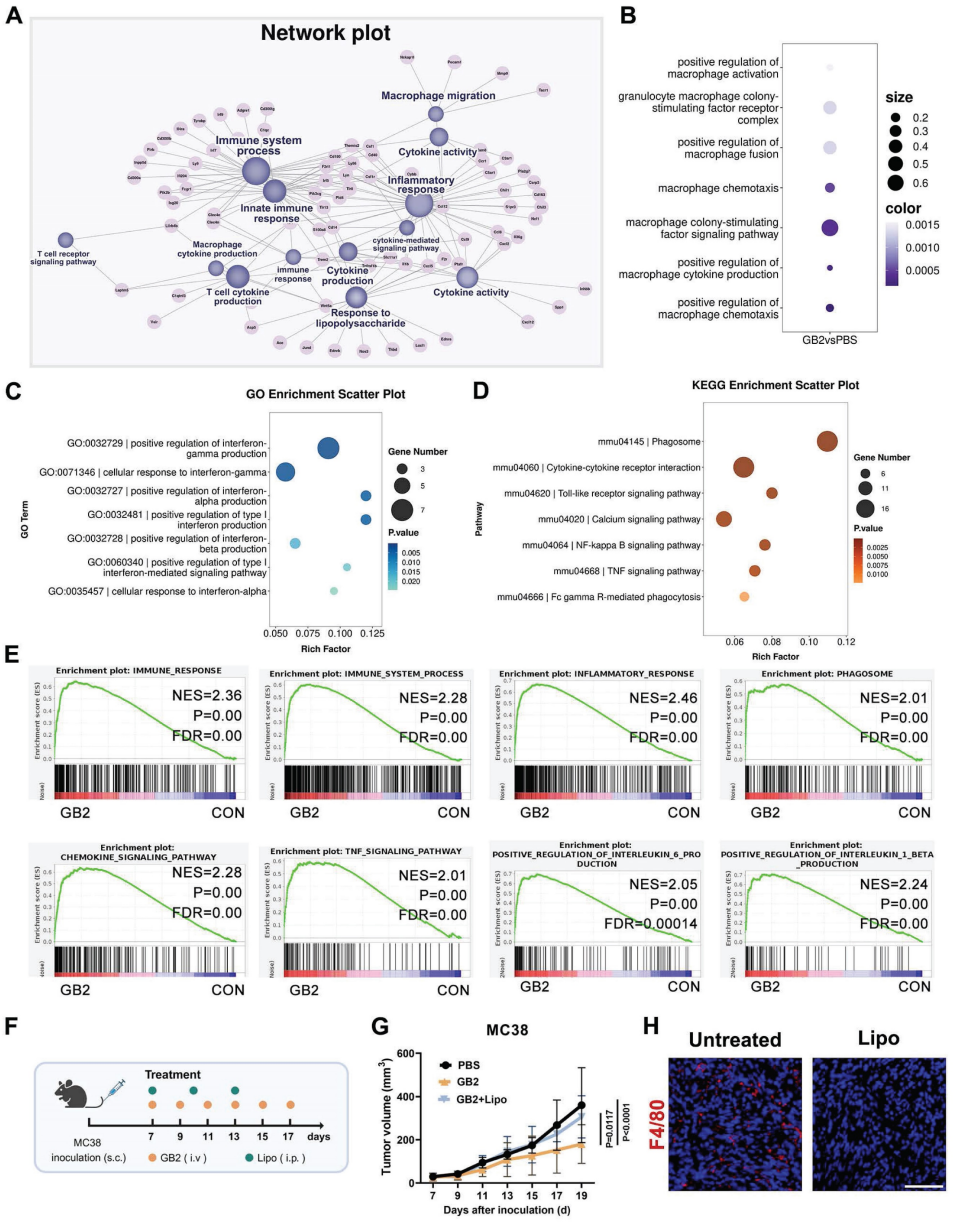
RNA-seq revealed that GB2 regulated macrophage to exert anti-tumor effect. (A) Network plot illustrating differentially expressed pathways and their correlated genes. Network plot was performed using the OmicStudio tools at https://www.omicstudio.cn/tool/56. Pink nodes indicate differentially expressed genes. Purple nodes indicate significant pathways. Lines connected nodes represent the subset correlation. (B) Dot plots for macrophage related pathways from GO enrichment, where dot size indicates enrich factor, color represents P value. (C) GO enrichment scatter plot for interferon-related pathways in RAW264.7 cells between GB2-treated and untreated groups. (D) KEGG enrichment scatter plot for pathways associated with macrophage function in RAW264.7 cells between GB2-treated and untreated groups. (E) GSEA plots for pathways significantly changed in RAW264.7 cells between GB2-treated and untreated groups. (F) *In vivo* macrophage depletion experiments: C57BL/6 mice were subcutaneously inoculated with MC38 tumors and treated with GB2 (10 mg/kg) intravenously and Lipo (200 μL/mouse) intraperitoneally. (G) Growth curve of MC38-inoculated mice with PBS treatment, GB2 treatment, and combinational treatment of GB2 and Lipo (*n* = 6). (H) IF images for macrophage determination via staining F4/80 expression in tumors. Scale bars: 100 μm. *n* indicates biological replicates. Error bars represent means ± SD. Differences between groups were tested using one-way ANOVA followed by Tukey's multiple comparisons test.

To elucidate the molecular mechanisms underlying the anti-tumor effects of prodrug GB2 in the tumor microenvironment, we performed bulk RNA-sequencing (RNA-seq) on untreated and GB2-treated MC38 tumors. Initially, we generated a network plot based on Gene Ontology (GO) enrichment analysis, where differentially expressed genes were represented as pink nodes and GO pathways as purple nodes, connected by dashes (Figure 3A). Remarkably, immune processes, inflammatory responses, and innate immune responses exhibited the highest centrality among all pathways, suggesting that GB2 plays an immune-modulating role. Cytokine-related pathways contribute a lot, including cytokine production, cytokine activity, cytokine-mediated pathway, T cell cytokine production, and macrophage production (Figure 3A). Macrophage-related pathways also show significant change, including macrophage colony-stimulating factor signaling pathway, macrophage chemotaxis, macrophage cytokine production, and macrophage activation (Figure 3B). Moreover, the enrichment of “response to lipopolysaccharide” pathway indicates a classical M1-like macrophage activation (Figure 3A).

While specifically focusing on macrophage-mediated cytokine production from GO analysis, key pathways concentrated in interferon alpha, interferon beta, and interferon gamma (IFN-γ) (Figure 3C). KEGG enrichment results identified pathways related to macrophage function and intrinsic signal transduction, including phagosome, cytokine-cytokine receptor interaction, Toll-like receptor signaling pathway, calcium signaling pathway, NF-kappa B signaling pathway, TNF signaling pathway, lysosome, and Fc gamma R-mediated phagocytosis (Figure 3D). Based on the above results, we inferred that macrophages are the main contributors in GB2-mediated anti-tumor immune response.

Gene set enrichment analysis (GSEA) was also conducted, in case that certain genes with minor fold change were missed, revealing significantly enriched pathways related to macrophage activation in the GB2 group, characterized by higher normalized enrichment score (NES) values and minor P values. Except for the pathways mentioned above, including immune process, immune system process, inflammatory response, phagosome, and chemokine signal pathway, we also found significant enrichment in pathways associated with inflammatory factor production such as TNF signaling pathway, regulation of interleukin-6 (IL-6) production (Figure 3E). Collectively, these findings establish that GB2 treatment induces an inflammatory response and activates the immune system in tumors, along with macrophage activation and macrophage-mediated cytokine signaling regulation.

To ascertain whether macrophages are the predominant contributors to the immune response, a macrophage depletion assay was performed by intraperitoneally injecting clodronate liposomes (Lipo) in mice (Figure 3F). Results revealed a significant difference in tumor burden between GB2-treated mice with or without Lipo administration (Figure 3G). For details, macrophage depletion abrogated the anti-tumor efficacy of GB2, emphasizing its role as a macrophage-dependent therapeutic drug. Additionally, we performed IF staining to determine macrophage existence in tumor tissues. There is no positive signal for F4/80 expression in Lipo group (Figure 3H). Given the crucial role of macrophages in antigen presentation and T cell immune activation, modulating macrophages with GB2 to enhance the anti-tumor immune response presents a promising approach for tumor therapy.

### GB2 inhibits TREM2 and activates *p*-STING in macrophages

**Figure 4:**
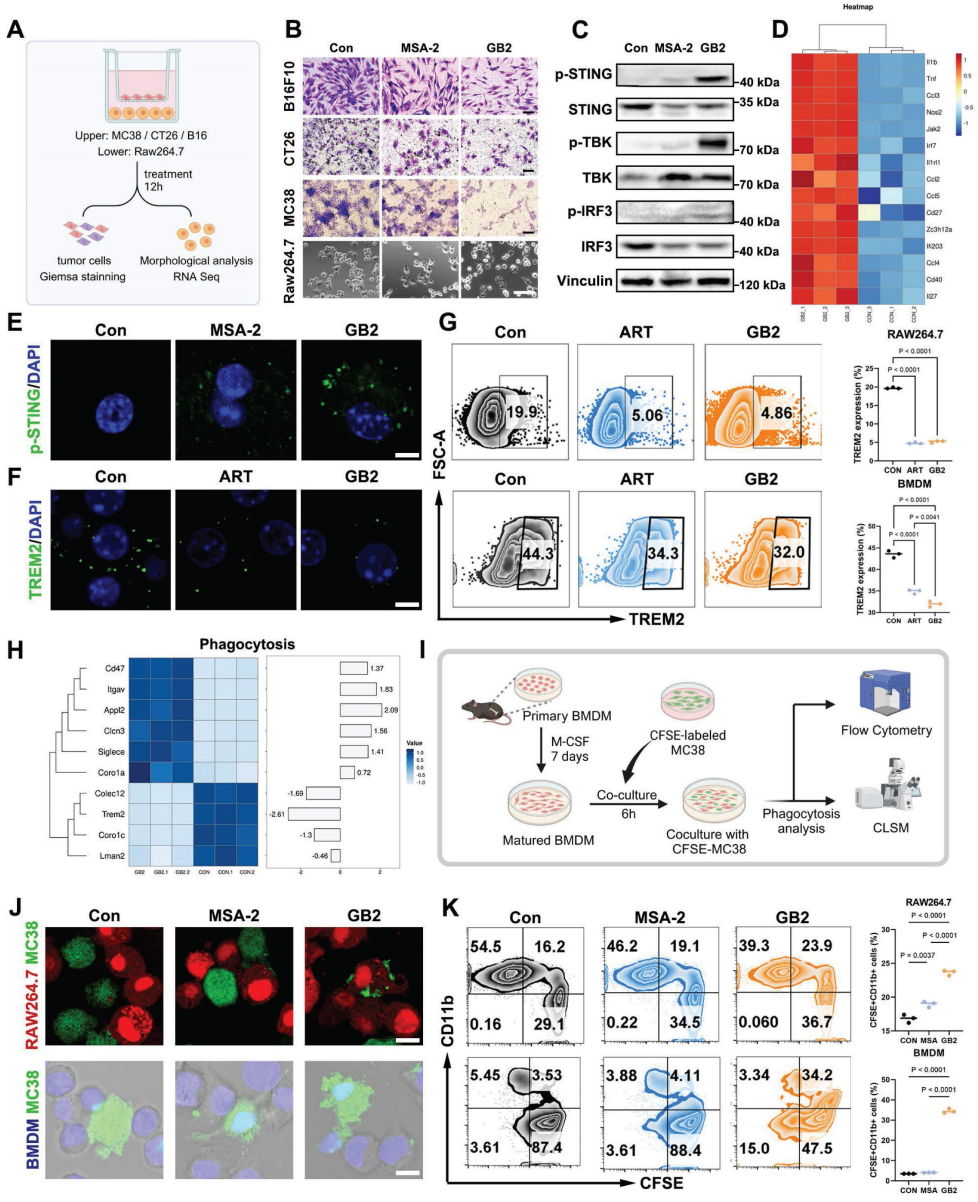
GB2 inhibits TREM2, activates *p*-STING, and enhances phagocytosis capacity in RAW264.7 and BMDM. (A) Experimental design: RAW264.7 cells (lower chamber) are cocultured with tumor cells (upper chamber), including MC38, CT26, and B16F10 in a transwell system, and treated with 10 μM MSA-2 and 10 μM GB2 for 12 hours. RNA-Seq was conducted to identify gene change in GB2-treated macrophages. (B) Microscopy observation for Giemsa staining of tumors and morphological analysis of RAW264.7 cells. Scale bars: 100 μm. (C) Western blot results for STING signaling cascades. Vinculin is set as a loading control. (D) Heatmap depicting differential expression of ISGs between untreated or GB2 treated RAW264.7 cells, normalized by z-score. (E) IF staining images for *p*-STING expression in BMDMs, treating with 10 μM MSA-2 and 10 μM GB2 for 12 hours. Scale bar: 5 μm. (F) IF staining image for TREM2 expression in BMDMs, treating with 10 μM ART and 10 μM GB2 for 12 hours. Scale bar: 5 μm (G) FCM for detecting TREM2 expression in BMDMs, treating with 10 μM ART and 10 μM GB2 for 12 hours. Quantitative analysis was conducted by calculating the positive rates of in indicated groups (*n* = 3). (H) Heatmap showing differential expression of genes related with phagocytosis between untreated or GB2 treated RAW264.7 cells, with values normalized by z-score (*n* = 3). (I) Experimental design: primary BMDMs were isolated from mouse bone marrow and stimulated with M-CSF (10 ng/mL) for 7 days. Matured BMDMs were cocultured with CFSE-labeled MC38 for 6 hours, treating with 10 μM MSA-2 and 10 μM GB2. Phagocytosis analysis was conducted by FCM and CLSM. (J) IF staining images for phagocytosis in untreated, MSA-2 treated, and GB2-treated mCherry-RAW264.7 cells (red) or BMDMs (blue), coculturing with CFSE-labeled MC38 (green). Scale bar: 10 μm. (K) FCM graphs for phagocytosis. MC38 was labeled with CFSE, and macrophages were stained with PercpCy5.5. Quantitative analysis was conducted by calculating the positive rates of CFSE^+^CD11b^+^ cells in indicated groups (*n* = 3). *n* indicates biological replicates. Error bars represent means ± SD. Differences between groups were tested using one-way ANOVA followed by Tukey's multiple comparisons test.

Having identified macrophages as the dominant target of GB2, we investigated how STING agonist GB2 influences TAMs utilizing the mouse monocyte-macrophage cell line RAW264.7 and mouse bone marrow-derived macrophages (BMDMs). To assess the potential of GB2-treated macrophages on tumor killing, we implemented a transwell co-culture system with tumor cells in the upper chamber and macrophages in the bottom chamber. Following a 12-hour treatment period, upper tumor cells were stained with Giemsa to identify cell proliferation, while the morphology of the bottom macrophages was evaluated by microscope (Figure 4A).

GB2-treated macrophages effectively inhibited the proliferation of B16F10, CT26, and MC38 cells in the co-culture system (Figure 4B). Meanwhile, both MSA-2 and GB2-treated macrophages exhibited a polarization towards a classical M1-like morphology characterized by multi-antennae morphology and increased pseudopod. Upon exploring the STING-involved mechanism, it was revealed that GB2 upregulated the phosphorylation level of STING signaling pathway cascades, including TBK and IRF in RAW264.7 cells (Figure 4C and Figure S8A-F). We also observed a precise colocalization of *p*-STING and F4/80 in GB2-treated tumors, suggesting the activation of STING signaling on macrophages *in vivo* (Figure S8G). To comprehensively investigate gene change in the bottom macrophages, we conducted RNA-seq. The differential gene heatmap displayed a comprehensive upregulation of interferon-stimulated genes (ISGs), including* Il1b, Tnf, Ccl3, Nos2, Jak2, Irf7, Ccl2, Ccl5, Cd27, Ccl4, Cd40, Il27, Ifi203, Zc3l12a, Il1h12a, etc* (Figure 4D). Such activation on ISGs indicates a comprehensive activation on STING signaling pathway in GB2-treated macrophages, consistent with the results of IF staining on *p*-STING in BMDMs (Figure 4E). ISGs are highly involved in multiple immune processes including inflammatory response, signal transduction, leukocyte migration, chemotaxis, etc. As a consequent result of STING activation, the upregulation on ISGs suggests that GB2-treated macrophages have changed significantly.

Verification of TREM2 inhibition was also conducted at both the gene and protein levels. RNA-seq result reveals a significant downregulation of *Trem2* gene expression and Western blot results confirmed a significant reduction in TREM2 protein levels (Figure S8H-J). GB2 effectively reduced TREM2 expression in RAW264.7 and BMDMs, quantified through flow cytometric analysis and visualized by IF staining. Consistent with ART treatment, GB2 maintained an equal or lower level of TREM2 expression compared to the Control group (Figure 4F-G).

DNAX-activation protein 12 (DAP12) is a transmembrane adapter that pairs with TREM2 and instigates TREM2-mediated protein phosphorylation signaling 32. Therefore, a block on TREM2 expression would impact DAP12 signaling transduction. RNA-seq data reveals that differentially expressed genes were significantly enriched in DAP12 interactions and DAP12 signaling with quite higher NES value and lower FDR value (Figure S8K, L).

### GB2 enhances phagocytosis capacity in macrophages

STING activation in macrophages tends to elicit an enhanced function on phagocytosis and antigen presentation 21. Given that genes related to phagocytosis changed significantly in GB2-treated RAW264.7 cells, we then investigated whether GB2 can intensify the phagocytotic capacity of macrophages compared to MSA-2 (Figure 4H).

CFSE-labeled MC38 tumor cells treated with GB2 or MSA-2 were co-cultured with RAW264.7 or BMDMs. After six hours, the cell mixture was collected, stained with CD11b-PercpCy5.5 antibody, and analyzed by flow cytometry (FCM) and confocal laser scanning microscope (CLSM) to determine the phagocytosis rate (Figure 4I). RAW264.7-mCherry cells were co-cultured with CFSE-labeled MC38 cells, treated or not, and we observed that GB2-treated macrophages engulfed tumor cells and caused their lysis (Figure 4J). Although MSA-2-treated macrophages migrated to tumor cells, they did not counteract, allowing tumor cells to survive. Parallel observations on phagocytosis were made when labeling BMDMs with the nucleic dye Hoechst 33342 (Figure 4J). Quantification of the phagocytotic rates reveals that GB2 significantly increased the portion of the CFSE^+^CD11b^+^ population from 3.53% to 34.2% in BMDMs, as shown in Figure 4K, with similar results in the RAW264.7 model.

Above all, we have demonstrated that GB2 can activate STING signaling cascades, downregulate TREM2 expression and its downstream pathways, and mutually enhance macrophage phagocytosis capacity in RAW264.7 and BMDMs.

### GB2 upregulates antigen presentation and initiates inflammatory response in macrophages

**Figure 5:**
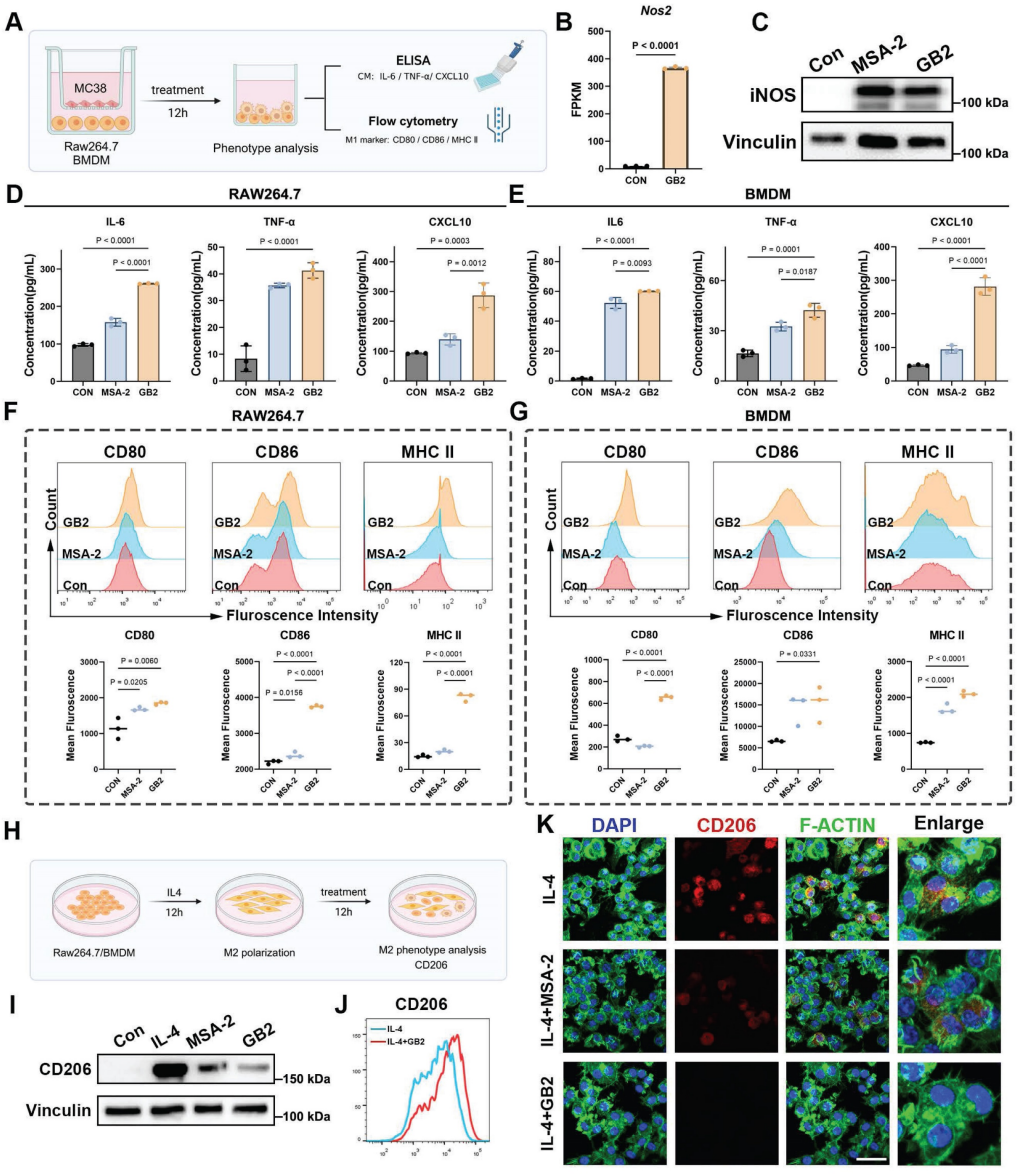
GB2 promotes M1-like macropahge phenotype by upregulating antigen presentation and initiating inflammatory response, and reverses IL4-induced M2-like macrophage phenotype. (A) *In vitro* experiments for macrophage phenotype analysis after treatment: BMDMs or RAW264.7 are cocultured with MC38 tumor cells in a transwell system, treating with 10 μM MSA-2 and 10 μM GB2 for 12 hours. CM was collected for ELISA to measure the content of IL-6, TNF-α, and CXCL10. BMDMs or RAW264.7 were collected for detecting M1 markers, including CD80, CD86, and MHC II, by FCM. (B) Gene expression of *Nos2*. (C)Western blot for macrophage activation marker iNOS in untreated, MSA-2 treated, and GB2 treated BMDMs. (D and E) Determination on content of IL-6, TNF-α, and CXCL10 in CM of untreated, MSA-2 treated, and GB2 treated RAW264.7 and BMDMs (n = 3). (F and G) Histograms of FCM detection on CD80, CD86, and MHC II in untreated, MSA-2 treated, and GB2 treated RAW264.7 and BMDMs. Quantitative analysis was conducted by calculating mean fluorescence in indicated groups (n = 3). (H) *In vitro* experiments for establishing M2 macrophages by IL4 stimulation: RAW264.7 or BMDMs were stimulated by IL4 (20 ng/mL) for 12 hours and treated with 10 μM MSA-2 and 10 μM GB2 for another 12 hours for phenotype analysis, employing Western blot, FCM and CLSM. (I) Western blot detection for CD206 expression in RAW264.7 cells treated with MSA-2 or GB2, VINCULIN is set as the loading control. (J) FCM was utilized to determine the reverse effect of GB2 on IL4 stimulation, by determining the expression of CD206 in RAW264.7 cells. (K) Representative IF images of IL4 treated, IL4 + MSA-2 treated, and IL4 + GB2 treated macrophages, staining with CD206 (M2 marker), F-ACTIN (cytoskeleton), and DAPI (nuclei). Scale bar: 30 μm. n indicates biological replicates. Error bars represent means ± SD. Differences between groups were tested using one-way ANOVA followed by Tukey's multiple comparisons test.

Continuing our investigation, we examined the antigen presentation function and inflammatory response of macrophages (Figure 5A). Nitric oxide synthase (iNOS), a crucial marker for macrophage inflammatory response, was assessed 22. The *Nos2* gene expression in GB2-treated macrophages displayed a similar upregulation trend (Figure 5B). Western blot results demonstrated an upregulation of iNOS in BMDMs after treatment with GB2 (Figure 5C and Figure S9A). Additionally, key effector molecules driving macrophages toward an inflammatory phenotype (IL-6, TNF-α, and CXCL10) were examined in the co-culture medium. The levels of these macrophage activation-related molecules surged in the culture medium after treatment, indicating that GB2 promotes macrophage to produce an inflammatory response (Figure 5D-E).

Antigen presentation capacity correlates with macrophage-mediated anti-tumor response. FCM was employed to detect surface co-stimulators CD80, CD86, and major histocompatibility complex II (MHC II), which are deemed markers of antigen-presenting macrophages (Figure 5A). Results showed that GB2 significantly upregulates CD80, CD86 and MHC II expression in both RAW264.7 and BMDM, providing quantitative analyses (Figure 5F-G). In addition, knocking down TREM2 gene from RAW264.7 macrophages blunted CD86 expression, indicating a reverse on phenotype. (Figure S9B-C). Collectively, GB2 drives an M1-like phenotype by eliciting inflammatory responses and enhancing antigen presentation capacity.

### GB2 reverses IL4-induced M2-like macrophage phenotype

In the context of TAMs predominantly exhibiting the M2 phenotype with pro-tumorigenesis properties, we explored whether GB2 could reverse M2-like macrophages towards an M1-like phenotype. IL4 stimulation was employed to induce M2-like macrophages *in vitro*, and this process was interrupted by treating with GB2 or MSA-2 (Figure 5H). Western blot and FCM results demonstrated that IL4 stimulation indeed led to a typical M2 phenotype with the highest expression of CD206, and this effect was significantly counteracted by the addition of GB2 (Figure 5I-J and Figure S9D-E). Although MSA-2 had a limited effect on this reversing process, we observed a stronger downregulation on CD206 level in the GB2-treated group (Figure 5I). IF staining was performed to further characterize morphological features. Cytoskeleton staining revealed that IL4 stimulation shaped spindle-like macrophages, while GB2 treatment completely prevented this process, resulting in a classical M1 morphology with irregular polygons (Figure 5K). Consistent with the morphological changes, CD206 fluorescence intensity also showed downregulation after GB2 and MSA-2 treatment.

Above all, our findings demonstrated that GB2 is a potent macrophage activator with the potential to reverse M2-like pro-tumor macrophages *in vitro*.

### Transcriptome analysis revealed that GB2 activates HIF-Glycolysis pathway and promotes mitochondrial fission

**Figure 6:**
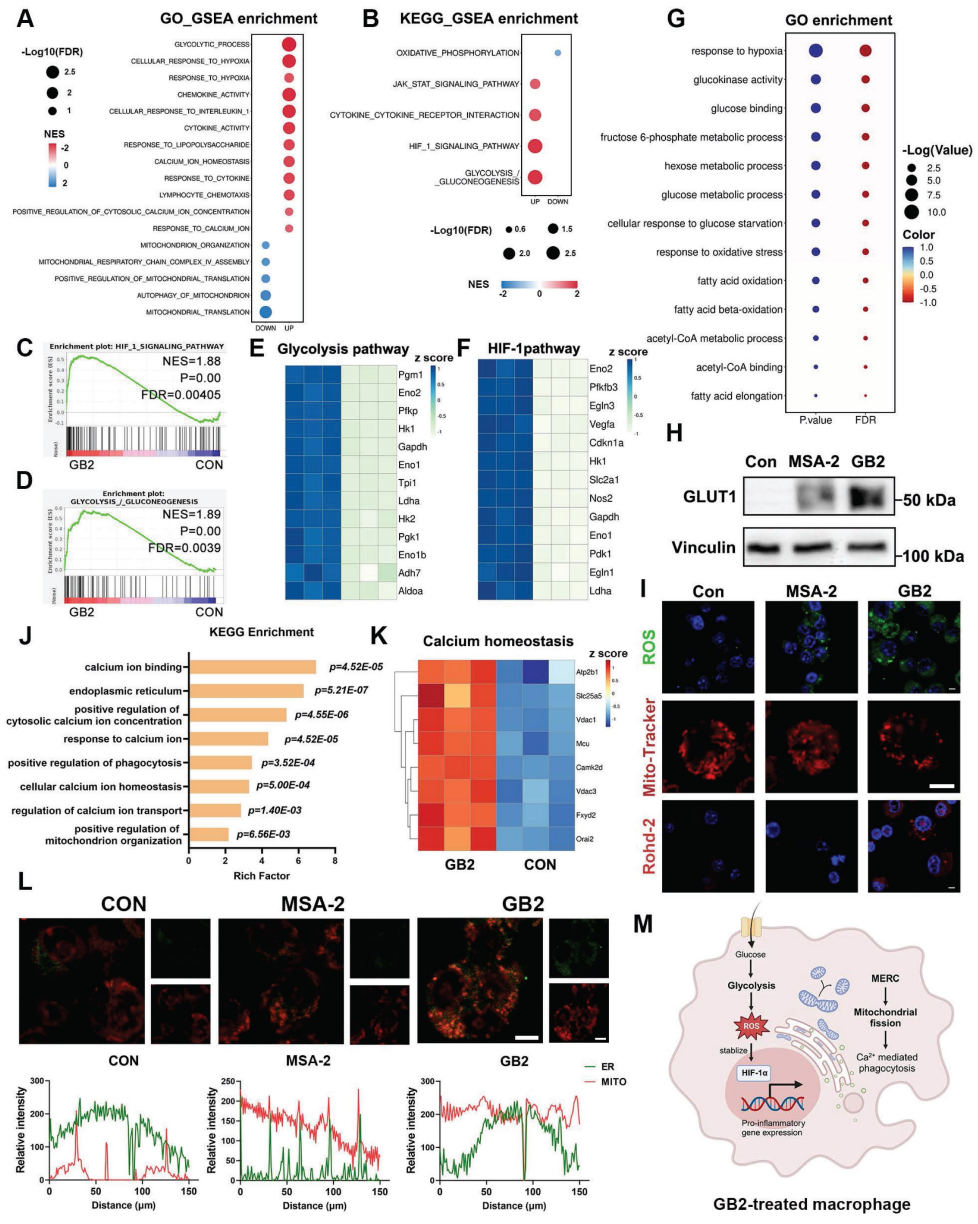
Mechanism analysis for GB2-treated macrophages. (A and B) GSEA analysis from GO (A) and KEGG (B) enrichment from RNA-Seq for untreated and GB2 treated RAW264.7 cells are listed by bubble chart, where the bubble size corresponds to the FDR value, and the bubble color reflects the NES value (n = 3). (C and D) GSEA analysis plot for HIF_1_SIGNALLING_PATHWAY (C) and GLYCOLYSIS_/_GLUCONEOGENESIS (D), displaying with NES value and P value (n = 3). (E and F) Heat map for genes related to glycolysis pathway (E) and HIF-1 pathway (F) between untreated and GB2 treated RAW264.7 cells, normalized by z-score. (G) Glucose-related pathways from GO enrichment are displayed by bubble chart, where P value and FDR are distinguished by color, and the value reflects bubble size (n = 3). (H) Western blot for the expression of GLUT1 in untreated, MSA-2-treated, and GB2-treated RAW264.7 cells. (I) Representative images of fluorescence staining for untreated, MSA-2-treated, and GB2-treated RAW264.7 cells, staining with ROS detector DCFH-DA, mitochondrial detector Mito-Tracker, and Ca^2+^ probe Rohd-2. Scale bars, 5 μm. (J) Ca^2+^ related pathway from KEGG enrichment displayed with a column chart, where x-axis represents rich factor, y-axis represents pathway names. (K) Heat map for genes related with calcium homeostasis between untreated and GB2 treated RAW264.7 cells, normalized by z-score (n = 3). (L) Representative images for determination on MERC, staining with Mito-Tracker (red) and ER-Tracker (green), in untreated, MSA-2-treated, and GB2-treated RAW264.7 cells. Intensity charts are displayed with x-axis for distance, y-axis for fluorescence intensity. Scale bars, 5 μm. (M) Schematic illustration for the reprogramming mechanism of GB2-educated macrophage. Treatment: 10 μM MSA-2 and 10 μM GB2 for RAW264.7 cells. n indicates biological replicates.

To further elucidate the mechanism of GB2 on activating macrophages, we conducted a comprehensive analysis of RNA-seq data in RAW264.7 cells, identifying a total of 2895 upregulated and 1351 downregulated genes in GB2-treated macrophages compared to untreated macrophages (Figure S10A-B). After analyzing these genes by GSEA in GO and KEGG databases, the results were displayed as bubble charts, where the bubble size corresponds to the FDR value, and the bubble color reflects the NES value. Consistent with the previous findings, responses to cytokine and leukocyte chemotaxis were upregulated (Figure 6A). Meanwhile, classical macrophage activation pathways, including the JAK-STAT pathway and cytokine-cytokine receptor interaction, were significantly upregulated (Figure 6B). These findings support that GB2 activates a pro-inflammatory phenotype in macrophages at the gene level.

Glycolytic process pathway and glycolysis/gluconeogenesis pathway emerged among all the pathways enriched in GO and KEGG pathways. The upregulated glycolytic process tends to produce massive reactive oxygen species (ROS), subsequently stabilizing the expression of HIF-1α, which positively regulates pro-inflammatory cytokine and glycolysis-related gene transcription 33, 34. Hence, an active glycolytic process is closely associated with HIF-1α. GSEA analysis and heatmap revealed a significant upregulation of genes related to glycolysis and HIF-1α after GB2 treatment (Figure 6C-F). It is well-documented that macrophages can reprogram their metabolic phenotype to meet various survival demands 34. Generally speaking, M1-like phenotype macrophages, which consume considerable amounts of glucose to fuel the inflammatory process, preferentially utilize glycolysis-based metabolism over oxidative phosphorylation (OXPHOS) 8. The downregulation on OXPHOS and the upregulation of glycolysis, as well as HIF-1α, suggest a shift in the metabolic phenotype of macrophages.

GO enrichment analysis further supported this transformation, with pathways related to glucose metabolism and glycolysis standing out with extremely large P and Q values, as opposed to OXPHOS and its downstream metabolic processes (Figure 6G and Figure S10C). Bubble charts illustrate the rankings of -log (P value) and -log (Q value) independently, with different group names distinguished by colors. Serving as gatekeeper for glycolysis, glucose transporter 1 (GLUT1) was significantly upregulated, indicating an enhanced glucose uptake fueling the glycolytic process (Figure 6H and Figure S10D). Consequently, ROS, an essential product of glucose metabolism, was detected through CLSM, revealing an upregulation in GB2-treated macrophages (Figure 6I). Moreover, the elevated lactic acid concentration in the culture medium verifies the higher glycolytic capacity of GB2-treated macrophages (Figure S10E).

Additionally, GB2 treatment downregulated pathways related to mitochondria, including mitochondrial translocation, organization, and autophagy (Figure 6A). Consequently, we analyzed mitochondrial status with Mito-Tracker, and results showed that GB2 reorganized mitochondria morphology from a filiform to a spherical form, indicating an upregulation on mitochondrial fission (Figure 6I). Mitochondrial fission also regulates calcium (Ca^2+^)-mediated phagocytosis in macrophages 35. KEGG enrichment data indicated that pathways involving Ca^2+^ metabolism, phagocytosis, and mitochondrial organization are significantly enriched (Figure 6J). Gene differential expression of Ca^2+^ homeostasis was illustrated in the heatmap (Figure 6K). Consistently, utilizing the Rohd-2 detector to determine calcium content, we found an elevation following GB2 treatment (Figure 6I). So far, GB2 induces mitochondrial fission to drive Ca^2+^-mediated phagocytotic process in macrophages.

Tracing the upstream mechanism of mitochondrial fission and Ca^2+^ flux, we assumed MERC, which was reported to be highly involved in M1 macrophage activation, as the main cause 36. To visualize MERC, mitochondria and endoplasmic reticulum (ER) were labeled with fluorescence probes and observed through CLSM. As Figure 6L depicted, an overlap of green (ER) and red (mitochondria) fluorescence was found in GB2-treated macrophages, and co-localization analysis displayed the fluorescence intensity of indicated sites. As a matter of fact, MERC disrupts Ca^2+^ homeostasis in ER, and forces Ca^2+^ to flow into mitochondria and cytoplasm, thus leading to mitochondrial fission.

To conclude, GB2 upregulates glycolysis, produces massive ROS to stabilize HIF-1α, which promotes pro-inflammatory gene transcription to support macrophage inflammatory response. Simultaneously, GB2 initiates MERC to promote mitochondrial fission, augmenting Ca^2+^-mediated phagocytosis (Figure 6M).

### GB2 reprograms tumor-associated macrophages *in vivo*

**Figure 7:**
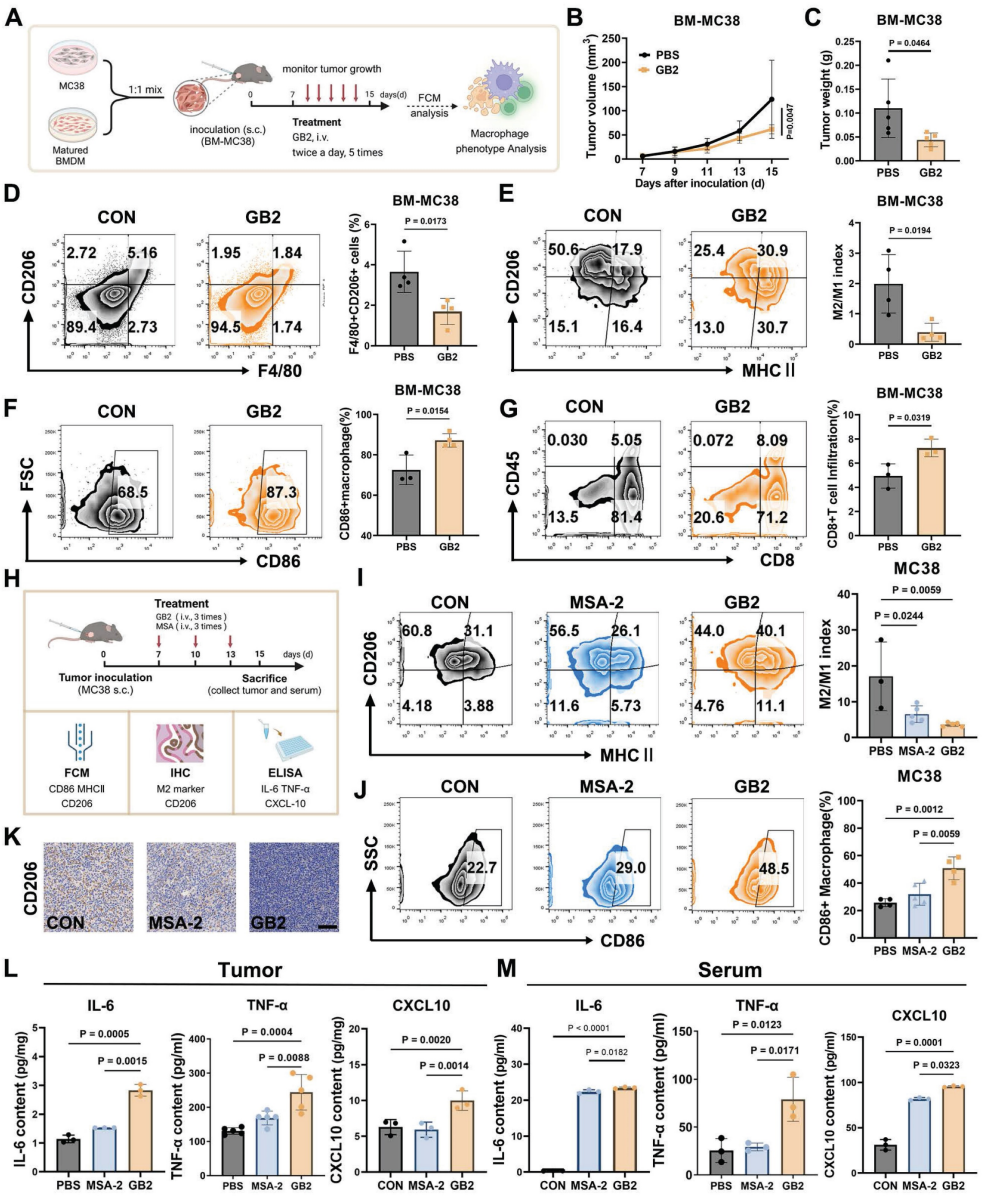
GB2 reprograms TAMs in murine colon tumor MC38 models. (A) Experimental establishment for TAMs phenotype characterization in BM-MC38 model: Matured BMDMs and MC38 tumor cells were mixed at a ratio of 1:1. The mixture was inoculated in C57BL/J mice. Seven days later, the mice were treated with GB2 (10 mg/kg) intravenously three times, and tumor volumes were recorded. FCM was employed to analyze the phenotype of TAMs. (B and C) Growth curve and tumor weight for BM-MC38 tumors (n *=* 5). (D) Frequency of TAMs (CD206^+^F4/80^+^ cells) in PBS and GB2-treated mice (n = 4). (E) Analysis of TAMs phenotype in PBS and GB2-treated tumor, staining with CD206 and MHC II and calculating with M2/M1 index (n = 4). (F) CD86 expression on TAMs in PBS and GB2-treated tumor (n = 3-4). (G) Infiltration of CD8^+^ TILs in BM-MC38 tumors (n = 3). (H) Experimental establishment for TAMs phenotype characterization and activation analysis in MC38 model: mice bearing with MC38 tumors were treated with PBS, MSA-2 (4 mg/kg), and GB2 (10 mg/kg) three times. On day 15, the mice were sacrificed, tumors and serum were collected. FCM and IHC were employed to determine phenotype markers, ELISA was conducted to detect cytokines related to macrophage activation. (I) Analysis of TAMs phenotype in PBS-treated, MSA-2-treated, and GB2-treated tumors, staining with CD206 and MHC II and calculating with M2/M1 index (n = 3-5). (J) CD86 expression on TAMs in PBS and GB2-treated tumor (n = 4-6). (K) Representative images of PBS-treated, MSA-2-treated, and GB2-treated tumors staining with CD206. Scale bar, 100 μm. (L and M) Content of macrophage activator, IL-6, TNF-α, and CXCL10, in both tumor and serum from mice treated with PBS, MSA-2, and GB2 (n = 3). *n* indicates biological replicates. Error bars represent means ± SD. Differences between groups were tested using one-way ANOVA followed by Tukey's multiple comparisons test.

To further investigate the impact of GB2 on TAMs *in vivo*, we created a TAM-enriched tumor microenvironment, as depicted in Figure 7A, by subcutaneously inoculating a mixture of MC38 tumor cells and BMDMs into C57 mice at a 1:1 ratio (BM-MC38 model). After treatments with GB2, we observed a significant inhibition of tumor growth and a reduction in tumor weight, indicating an extensive regression in the TAM-enriched tumor (Figure 7B-C).

The analysis of cell populations in BM-MC38 tumors revealed that in the PBS group, macrophages maintained a higher F4/80^+^ CD206^+^ population at 5.18%, indicating a transformation of BMDMs into M2-like TAMs. On the contrary, GB2 treatment significantly shrunk F4/80^+^ CD206^+^ population to 1.84% among whole cells. Further characterization of TAM phenotype was conducted by defining the M2/M1 index as the ratio of MHC II^+^CD206^-^ portion to the MHC II^-^CD206^+^ portion. Results demonstrated that GB2 decreased CD206 expression, increased MHC II expression, resulting in a significantly lower M2/M1 index, indicating a comparable reversal of the M2-like macrophage phenotype (Figure 7E). Meanwhile, the M1 marker CD86 maintained a higher level in macrophage from GB2 treated tumor (Figure 7F). The increase on the amount of CD8^+^ TILs suggested that GB2 effectively reprogrammed macrophages towards an antigen-presenting phenotype (Figure 7G).

Inspired by the above results, we conducted experiments in a pure MC38 tumor model, using MSA-2 as a positive control, to corroborate the efficacy of GB2 in TAM reprogramming *in vivo*. The expression of CD86 and CD206 as well as M2/M1 index were detected by FCM to identify TAM phenotype, while cytokines related to macrophage activation, including IL-6, TNF-α, and CXCL10, were determined in tumor tissue and serum after treatment (Figure 7H). For the M2/M1 index, macrophages in the GB2 treatment group exhibited an extremely lower level than the PBS group, with the portion of CD206^+^ macrophage decreasing from 60.8% to 44% and the portion of MHC II^+^ macrophages increasing from 3.88% to 11.1% (Figure 7I). Meanwhile, GB2 significantly increased CD86 expression in macrophages, suggesting an enhanced antigen-presenting function (Figure 7J). Additionally, IHC staining displayed that GB2 reduced overall CD206 expression in tumors (Figure 7K). Evaluation of macrophage activating cytokines showed that GB2 treatment indeed led to a significant upregulation of IL-6, TNF-α, and CXCL10 both in tumor and serum (Figure 7L-M). These results align with the *in vitro* conclusion that GB2 is an effective macrophage activator.

All these findings support the notion that GB2 reprograms TAMs towards an anti-tumor phenotype with enhanced effector function both *in vitro* and *vivo*.

### GB2 promotes CD8^+^ T cell infiltration, enhances T cell function, and improves αPD-1 therapy

Tumor-infiltrated CD8^+^ T cells are associated with improved prognosis and a more active response to immune checkpoint blockade (ICB) therapy 23. CD8^+^ T cells play a crucial role in orchestrating the overall immune response and directly killing tumor cells. The infiltration of CD8^+^ T cells into tumors is often hindered in many solid tumors due to the presence of pro-tumor TAMs 37. On the one hand, pro-tumor TAMs express high level CD206 and produce immunosuppressive cytokines, such as IL-10 and TGF-β, to directly or indirectly inhibit T cell proliferation and activation 8. On the other hand, increased lysosome activity in pro-tumor TAMs creates an acid intracellular environment, degrading antigen and impeding antigen cross-presentation to CD8^+^ T cells 38. Notably, RNA-seq data revealed a significant downregulation of lysosome activity in the GB2-treated group compared to the PBS group, suggesting that GB2-treated macrophages possess a stronger antigen-presentation capacity for T cell activation (Figure S10F).

**Figure 8:**
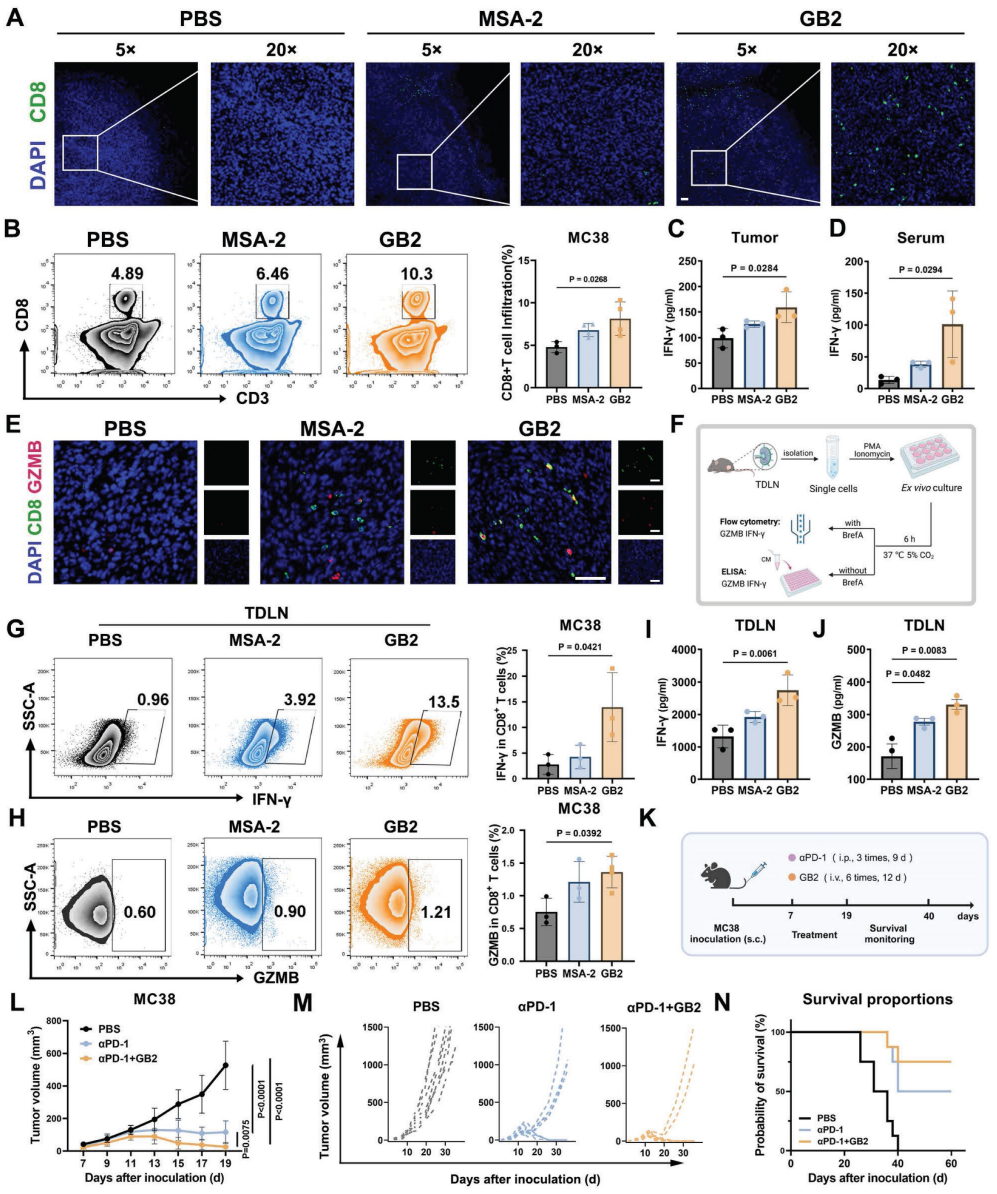
GB2 promotes CD8^+^ T cell infiltration and effector function, and augments the efficacy of αPD-1 therapy in MC38 mouse model. (A) Representative IF images of PBS-treated, MSA-2-treated, and GB2-treated tumors at day 8 after treatment, staining with CD8 (green) and DAPI (blue). Scale bars, 50 μm. (B) Representative FCM graphs and quantitative analysis of CD8^+^ T cell infiltration in PBS-treated, MSA-2-treated, and GB2-treated tumors at day 8 after treatment, staining with CD3, CD8 in CD45^+^ living lymphocytes (n = 3-4). (C and D) Content of IFN-γ in tumor and serum from PBS-treated, MSA-2-treated, and GB2-treated tumors at day 8 after treatment (n = 3). (E) Representative IF images of PBS-treated, MSA-2-treated, and GB2-treated tumors at day 8 after treatment, stained with GZMB (red), CD8 (green), and DAPI (blue). Scale bars, 50 μm. (F) Experimental design: TDLNs were isolated from PBS-treated, MSA-2-treated, and GB2-treated C57BL/6 mice, the single cell suspensions were treated with 100 nM porbol 12-myristate 13-acetate (PMA) and 500 ng/mL ionomycin, adding Brefeldin A (BrefA) or not, for six-hour *ex vivo* re-stimulation. FCM was employed to determine intracellular content of IFN-γ and GZMB. ELISA was utilized to determine the extracellular concentration of IFN-γ and GZMB (G and H). Frequency of IFN-γ^+^ CD8^+^ T cells or GZMB^+^ CD8^+^ T cells after re-stimulation in single cell suspension of TDLNs from mice treated with PBS, MSA-2 and GB2 (n = 3-4). (I and J) Content of IFN-γ and GZMB in the CM of re-stimulated TDLN cells, determined by ELISA (n = 3). (K) Experimental for synergistic therapy: MC38-inoculated C57BL/6 mice were treated with GB2 (10 mg/kg) and αPD-1 (100 μg/mouse) when tumor volumes reach 50 cm^3^. Tumor volume and mouse survival rate were monitored (n = 8). (L) Synergistic efficacy of αPD-1 and GB2 compared to PBS-treated, PD-1-treated tumor. (M) Tumor growth curves for each group with indicates treatment (n = 8). (N) Survival of C57BL/6 mice bearing MC38 tumors after treatment with PBS, αPD-1or combination of αPD-1 and GB2 (n = 8). *n* indicates biological replicates. Error bars represent means ± SD. Statistical analyses were performed using the Log-rank (Mantel-Cox) test for survival analysis. Differences between groups were tested using one-way ANOVA followed by Tukey's multiple comparisons test.

To figure out whether GB2 can reverse CD8^+^ T cell restriction in tumors, IF staining was evaluated. Results demonstrate that treatment with MSA-2 or GB2 increases CD8 expression in tumors compared to the PBS group (Figure 8A). However, CD8 signals in the MSA-2 group are mostly localized at the tumor margin rather than the interior, indicating disabled migration and infiltration of CD8^+^ T cells. In contrast, GB2 promotes CD8 expression both at the margin and interior, potentially disrupting the "barrier" constructed by peritumoral TAMs that impedes CD8^+^ T cell infiltration (Figure 8A). FCM for single-cell analysis in tumor tissue further confirmed that GB2 treatment elevated the portion of CD8^+^ T cells from 4.89% to 10.3% of CD45^+^ lymphocytes, indicating that GB2 recruited a significant number of CD8^+^ T cells into the tumor and promotes their migration to the interior (Figure 8B-C).

Tumor-infiltrated CD8^+^ T cells are functionally impaired, to some extent, and exhibit “exhausted” phenotype, with decreased secretion on IFN-γ and the protease granzyme B (GZMB) 39. To study that whether GB2 could prevent T cell dysfunction, we evaluated effector molecules IFN-γ and GZMB in tumor, serum, and tumor draining lymph nodes (TDLN). Content of IFN-γ in tumor and serum both increases significantly after GB2 treatment (Figure 8D-E). Meanwhile, IF staining displays that GB2 increases number of GZMB^+^CD8^+^ cells in tumor, suggesting a stronger tumor killing capacity of infiltrated CD8^+^ T cell (Figure 8F). Likewise, GB2 treatment augmented effector potential of CD8^+^ T cells in TDLN, with higher ability on secreting GZMB and IFN-γ after *ex vivo* re-stimulation (Figure 8G-H). Lymph nodes are important immune organ where constantly transfer CD8^+^ T cells into inflammatory sites. It is reported that CD8^+^ T cells in TDLN have strong capability on preventing tumor metastasis. We believe such enhancement on CD8^+^ T cell number and function demonstrates GB2's potential on immune modulation.

The TDLN accommodate systemic CD8^+^ T cell responses, which are associated with the efficacy of α-PD-1 therapy 40. To characterize whether GB2 has potential to improve ICB therapy, we performed a combinational treatment of GB2 and αPD-1 on MC38-bearing mice (Figure 8K). Results demonstrated that GB2 improved sole αPD-1 therapy significantly, and prolonged mice survival period (Figure 8L-N). Noticeably, tumors from six of eight mice completely vanished in combinational group, while only four mice vanished from cancer in αPD-1 group.

In conclusion, GB2 promotes CD8^+^ T cells to infiltrate into tumor interior and enhances their effector function on secreting GZMB and IFN-γ. Synergistic therapy by combining GB2 with αPD-1 significantly improves tumor-bearing mice survival.

## Discussion

Immunotherapy has emerged as a promising treatment strategy for both orthotopic and metastatic CRC 41. TAMs constitute a large proportion in colon tumor tissues, and play a crucial role in tumor progression as well as immunosuppression 42, 43. STING agonists activate innate immune pathways by triggering the cGAS-STING signaling cascades and inducing IFN-Ⅰ production, resulting in a potent anti-tumor effect in CRC 44. However, the extensive expression of the cGAS-STING signaling pathway in various cell types, including DCs, macrophages, NK cells, and endothelial cells, raises concerns about non-selective cytokine storms during systemic administration of STING agonists 11, 45. Our prodrug GB2 presented aggregated stability and precise tumor response which makes GB2 suitable for intravenous injection. Our findings demonstrated the efficacy of GB2 on tumor regression in mouse models of MC38 and CT26 colon cancer, as well as in a STING^low^ mouse melanoma model.

TREM2, originally identified as a myeloid receptor sustaining microglial response in Alzheimer's disease, has garnered attention in recent years due to its documented expression in various tumors, including breast cancer, ovarian cancer, colon cancer, and sarcoma 46. Notably, its specific high expression in tumor tissues is linked to poor prognosis 47. In this work, we presented a novel approach involving a TAM-targeting STING agonist prodrug GB2 by incorporating the newly characterized TREM2 inhibitor ART. This targeted strategy aims to enhance the therapeutic benefits of STING agonists while minimizing the risk of systemic cytokine storms. Through RNA-seq analysis of GB2-treated tumors, we verified that macrophages played a pivotal role in mediating the anti-tumor effects of GB2. Additionally, macrophages depletion counteracts the anti-tumor effects of GB2.

TREM2-expressing TAMs promote tumor progression through immunosuppression, driving CD8+ TILs dysfunction and NK cell paucity 47-50. Our study leveraged ART to target TREM2 in TAMs, therefore activating their intrinsic STING signaling cascades in macrophages to restore the anti-tumor effect of macrophages and mutually reverse immunosuppression. At the cellular level, GB2-treated macrophages exhibited improvement on antigen presentation, inflammatory response, and phagocytosis than MSA-2. In mouse colon cancer models, GB2-treated macrophages successfully reversed immunosuppression via increasing CD8^+^ TILs infiltration and enhancing CD8^+^ TIL effector function.

Previously reported TREM2-targeting drugs encountered limitations related to antibody administration, characterized by uncontrolled side effects due to prolonged half-life and necessitating advanced development techniques 51, 52. ART, recognized as a TREM2 inhibitor, has exhibited effectiveness in selectively influencing tumor-associated monocytes by targeting TREM2, thereby eliciting an anti-tumor effect 25. Within our investigation, ART demonstrated its proficiency in downregulating TREM2 protein and gene expression, along with its downstream DAP12 signaling pathways in macrophages. As a prodrug encapsulating ART, GB2 consistently demonstrated the same inhibitory effect on TREM2.

Our study validated the feasibility of addressing these challenges by combining the TREM2 small molecule inhibitor ART with the STING agonist MSA-2 in the formulation of prodrug GB2. This combination demonstrated the ability to mutually modulate TAMs, resulting in tumor regression and immune activation. Notably, when GB2 was combined with αPD-1 therapy, a synergistic effect was observed, leading to significant inhibition of tumor growth and prolonged mouse survival. We also found therapeutical effects of GB2 on human colon cancer model in nude mice. These findings highlight the clinical translation potential of GB2 as an effective prodrug for enhancing anti-tumor immunity in the context of colon cancer treatment.

To explore the mechanism, RNA-seq analysis substantiate our prodrug design strategy that GB2 activated the STING signaling cascades and ISGs while concurrently inhibiting TREM2-associated pathways. Besides, we observed a significant upregulation of glycolysis and HIF-1α pathways alongside a downregulation of OXPHOS. This dual effect signifies a metabolic transformation, facilitating the phenotypic characterization of TAMs. Upregulation of GLUT1 indicates enhanced glucose uptake in macrophages, which may disrupt the tumor's nutritional supply, thereby impeding tumor growth. Such metabolism reprogramming provided an insight on TAM-targeting therapy.

Mitochondria serve as a pivotal platform for signal transduction, playing a crucial role in macrophage biology. Mitochondrial morphology also varies among macrophage phenotypes 53. For instance, spherical or fragmented mitochondria caused by mitochondrial fission are involved in LPS-activated macrophages 54. Our data suggest that GB2-downregulated genes are enriched in mitochondria-associated pathways, triggering mitochondrial fission. On the one hand, such mitochondrial morphology reflects the transformation to a pro-inflammatory phenotype. On the other hand, mitochondrial fission also connects with macrophage phagocytosis via regulating Ca^2+^ flux. Likewise, Ca^2+^ serves as a vital signaling molecule that coordinates intracellular and extracellular signal transduction during the process of inflammatory regulation in macrophages. We believe that such a reinforcing loop among mitochondrial fission, Ca^2+^ flux, phagocytosis, and the inflammatory response collectively enhances macrophage-mediated anti-tumor functions.

Organelle interaction constitutes a fundamental process in cellular functions 55. While the occurrence of MERC in tumor cells has been associated with cell death, MERC has been shown to support the metabolic fitness and function of CD8^+^ T cells 56, 57. However, the mechanisms by which MERC regulates macrophages have been seldom explored. In our study, we discovered that MERC occurred in GB2-treated macrophages, potentially serving as the origin for mitochondrial fission and contributing to Ca^2+^-mediated phagocytosis as well as the inflammatory response of macrophages. MERC-driven mitochondrial fission and its role in promoting Ca^2+^-mediated phagocytosis provide novel insights into the intricate interplay between organelle dynamics and macrophage anti-tumor functions.

In summary, our study presents a novel STING agonist prodrug, GB2, specifically designed to reprogram macrophages in the tumor microenvironment. By targeting the inhibitory receptor TREM2 and activating the STING signaling pathway in TAMs, GB2 demonstrates promising therapeutic effects in mouse colon cancer models. Enhancement in CD8^+^ T cell infiltration and effector function, along with the synergistic augmentation of αPD-1 therapy, highlights the potential clinical significance of GB2 in CRC immunotherapy.

Looking ahead, the development of GB2 opens avenues for the design of innovative prodrug strategies aimed at manipulating TAMs to reshape the tumor immune landscape. Though extraordinary anti-tumor effect has been proved in mouse models, there is still a long way for clinical application of GB2, which might be limited by tumor heterogeneity and the veiled long-term toxicity.

## Methods

### Study design

The objective of this study was to determine the therapeutic effect of prodrug GB2 on MC38 tumor and to explore the intrinsic mechanism of GB2 treatment in macrophages. We primarily figured out that prodrug GB2 activated STING signal pathways and downregulated TREM2 expression to inhibit tumor growth, then identified that prodrug GB2's effect depended on tumor associated macrophages. *In vitro*, we utilized RAW264.7 cells and primary BMDMs as research models to study the action mechanism in GB2-treated macrophages. For *in vivo* experiments, mice were randomly assigned to each group. No data were excluded from the analysis.

### Animals

6-week-old healthy BALB/C, C57BL/6, and BALB/C-NU female mice (weight, 18-20 g) were purchased from SPF (Beijing) Biotechnology Co., Ltd. All the animals were housed in a pathogen-free condition in groups of 5-6 mice per cage, and the temperature was kept at 21 ± 2 °C, and the relative humidity was maintained at 40-70% with a 12 h light/dark cycle. Before the experiment, fed these mice in the stable environment for at least 1 week. All the procedures followed the guidelines of the Institutional Animal Care and Use Committee at Northwestern Polytechnical University.

### Tumor models

For pharmacological evaluation: MC38 tumor cells (1×10^6^ cells/mouse in 50 μL DMEM medium) and B16F10 (5×10^5^ cells/mouse in 50 μL DMEM medium) were inoculated in the right flank of C57/BL6 mice. CT26 (1×10^6^ cells/mouse in 50 μL 1640 medium) were inoculated in the right flank of BALB/C mice. For BM-MC38 models, 2×10^5^ MC38 tumor cells and 2×10^5^ BMDMs were mixed and inoculated in the right flank of BALB/C mice. HCT116 (1×10^8^ cells/mouse in 50 μL 1640 medium) were inoculated in the right flank of BALB/C-NU mice.

### Cell lines

The murine colon cancer cell line CT26 (Cell Bank of the Chinese Academy of Sciences, China) was cultured in the complete RPMI-1640 medium (Sangon Biotech, Shanghai, E600028) including 10% fetal bovine serum (FBS, Excell Bio, Shanghai, FSP500) and 1% penicillin-streptomycin 100x (PS, Macgene, Beijing, CC004). The murine melanoma cell line B16F10, the murine colon cancer cell line MC38, and the murine monocyte-macrophage leukemia cell line RAW264.7 was purchased from Cell Bank of the Chinese Academy of Sciences and cultured in the complete DMEM medium (high glucose, Sangon Biotech, Shanghai, E600003) including 10% FBS and 1% penicillin-streptomycin 100x. RAW264.7-mCherry was purchased from Ubigene Biosciences. All the cells were incubated in their special medium and maintained in a cell culture box (ThermoFisher-Forma 371) with 5% CO_2_ at 37℃.

### BMDM extraction

Bone-marrow-derived macrophages (BMDMs) were extracted from 6-week-old female C57BL/6 mice and cultured in complete DMEM medium supplemented with macrophage colony-stimulating factor (M-CSF) (20 ng/mL, Sangon Biotech) at 37 °C with 5% (v/v) CO_2_. After 7 days, attached cells are deemed as BMDMs and collected for subsequent experiments.

### Characterization of GB2

The stability of GB2 in PBS (pH 7.4) and responsive release in PBS with 10 mM GSH were determined at 0h, 6h, 12h, and 24h by HPLC (Waters, e2695) with a mobile phase consisting of acetonitrile and H_2_O (95:5, v/v). Zeta potential and particle size were determined by phase analysis light scattering (Brookhaven 90 Plus PALS).

### Biodistribution of GB2

Prodrug GB2 was mixed with 1 mg/kg DiR dye to form a co-assembled mixture GB2@DiR. MC38 tumor cells (1×10^6^ cells/mouse in 50 μL DMEM medium) were inoculated in the right flank of C57/BL6 mice. After 10 days, GB2@DiR was injected in mice intravenously. Fluorescence was monitored at 0 h, 3 h, 6 h, 12 h, and 24 h by IVIS Spectrum, DiR treatment was set as control.

### FCM

For tumor tissues, we collected tumors and dissociated them with collagenase Ⅳ and DNase Ⅰ at 37 ℃ for 30 min. Then screen the tumor lysate with 70-μm filters and collect the single cell suspension. Count the cells and aliquot to indicate cell density, when stained with antibodies. For cell lines, cells were washed and collected for staining. All cells were blocked with CD16/32 antibodies for 30 min, and stained with surface antibodies. For intracellular antibodies, cells were primarily fixed with 4% PFA and stained with antibodies diluted by permeability buffer after blocking and surface staining. All the antibodies used in FCM are listed in Supplementary Table S1.

### Western blot

Cell samples were washed three times with cold PBS and lysed on ice for 30 min in RIPA lysis buffer with protease inhibitor cocktail and PMSF. The lysates were then centrifuged at 15000 rpm for 10 min at 4 ℃. The supernatant was collected and desaturated by loading buffer. Samples were then separated on 10 or 12% SDS-polyacrylamide gels and transferred onto 0.45-μm PVDF membrane. After 1 hour of shaking in blocking buffer (3% nonfat milk or BSA in PBS containing 0.05% Tween 20), membranes were incubated with primary antibodies overnight at 4 °C. Next day followed by three washes using PBST buffer (PBS with 0.05% Tween 20) for 5 min per each, the membrane was incubated with HRP-linked secondary antibody. After 1 hour, the membrane was reacted with ECL buffer, then exposed by chemiluminescence imaging system (VILBER-VILBER FUSION FX6.EDGE). All the antibodies used for Western blot were listed in Supplementary Table S2.

### Giemsa staining

After transwell coculturing, the upper chamber was washed with PBS for three times, and fix with 4% PFA for 10 min. Then, the chamber was soaked into Giemsa staining buffer for 10 min. After staining, the chamber was dried and recorded by microscopy.

### MitoTracker labeling

MitoTracker™ Orange (ThermoFisher Scientific, M7510) was used to detect mitochondria in living cells. After drug treatment, cells are washed with PBS and incubated with MitoTracker™ Orange (100 nM, 37 ℃, 30 min) to stain mitochondria. Finally, use the confocal microscope to observe mitochondria morphology.

### Intracellular ROS and Ca^2+^ detection

Intracellular ROS generation was measured by FCM and CLSM by using a fluorescent dye DCFH-DA (2 μM) for 30 min at 37 ℃. Content of Ca^2+^ was detected by Rhod-2, AM (5 μM), a cell permeable dye agent with CLSM.

### RNA-seq

Total RNA was collected by Trizol (ThermoFisher, 15596018) following the manufacturer's procedure and purified and measured by Bioanalyzer 2100 and RNA 6000 Nano LabChip Kit (Agilent, CA, USA, 5067-1511) high-quality RNA samples with RIN number > 7.0 were used to construct sequencing library. RNA-seq was performed by LC Sciences through the Illumina X10 platform (Hangzhou, Zhejiang, China). Genes with the parameter of false discovery rate (FDR) below 0.05 and absolute fold change≥2 were considered differentially expressed genes. Differentially expressed genes were then subjected to enrichment analysis of GO functions and KEGG pathways. RAWdata for the RNA-seq of *in vivo* MC38 tumors from GB2-treated mice and bulk RNA-seq of RAW 264.7 cells have been deposited in GEO (GSE251810, GSE251808).

### ELISA

Cell culture medium was centrifuged (10000 rpm) for 10 min, collect supernatant for assay. Tumor tissue was cut into pieces and lysed for 1 hour on ice. Serum sample was collected from coagulated blood after centrifuging (5000 rpm) twice at 4 ℃. All samples were assayed following the manufacturer's instructions. ELISA assay kits: IFN-α (ThermoFisher, BMS6027), IFN-β (R&D system, MIFNB0), IL-6 (Invitrogen, 88-7064-88), TNF-α (Invitrogen, 88-7324-88), CXCL10 (Invitrogen, BMS6018MST), IFN-γ (Invitrogen, 88-7314-88), GZMB (Invitrogen, 88-8022-88).

### ScRNA-Seq data processing

We utilized Seurat R package (version 4.1.0) and applied standard downstream processing for scRNA-seq data. Genes that detected in less than 3 cells as well as cells with less than 200 detected gene numbers were ruled out, and the mitochondria proportion was limited to less than 20%. We then identified highly variable genes for subsequent analysis, setting the highly variable genes number to 2000. We constructed cell clusters using the “FindClusters” and “FindNeighbors” functions, and visualized them using the “t-SNE” method. Finally, we performed cell annotation based on the marker genes of different cell types.

### H&E staining

Mouse skin was isolated and fixed with 4% PFA (Servicebio, G1101). After gradient dehydration, samples were soaked into paraffin and make 4 μm slices. Stain with nuclei dye hematoxylin for 10 min and wash with running water, then dip into hydrochloric ethanol for 20 s. After washing with ddH_2_O, tissue slides were immersed in eosin for 2 min to get cytoplasm counterstain. With one dip of water wash, slides went through dehydration process by soaking into 95% ethanol solution and 100% ethanol. Seal the sample with neutral balsam (Solarbio, G8590), and store at room temperature.

### IF staining

Immunofluorescence staining has been performed on cell and tumor tissue sections and observed with confocal microscopy. For cell samples, membrane protein was stained with primary antibody (4 ℃, 12 h) and incubated with secondary antibody, while intracellular protein was primarily fix and permeabilized in PBS with 0.1% Triton then stained as membrane protein. DAPI was employed to label nuclei. Tissue samples follow paraffin embedding and slicing procedure and stain with indicated antibody. All the antibodies used in this part can be found in Supplementary Table S2.

### IHC staining

Tumor was isolated from mice after treatment, and the protein expression of Ki67, CD206, and TREM2 was evaluated. Paraffin slides were blocked and stained with indicated antibodies. PBS buffer containing 3% H_2_O_2_ was used for blocking endogenous peroxidase. Then, DAB staining kit was used for signaling amplify, and hematoxylin was employed for nuclei staining. The information of Ki67, CD206, and TREM2 antibodies is listed in Supplementary Table S2.

### Phagocytic assay

MC38 tumors were cocultured with RAW264.7/BMDMs for 12-hour treatment. For FCM, MC38 tumor cells were labeled with CFSE, and macrophages were stained with CD11b-PercpCy5.5 to discern the cell population. The portion of CFSE^+^CD11b^+^ was deemed as phagocytosis. For CLSM imaging, mCherry-RAW264.7 cells were cocultured with CFSE-labeled MC38 tumors. BMDM was stained with Hoechst 33342 and cocultured with CFSE-labeled MC38 tumors.

### *Ex* vivo experiments

Tumor TDLNs were isolated from C57 mice and made into single-cell suspension. Centrifuge the suspension and count cell number, then suspend the cells with 1640 culture medium and transfer them into 12-well culture plates with a density of 10^5^ cells per well. PMA and ionomycin were added to stimulate cytokine production. Six hours later, secretory cytokines were determined through ELISA. For intracellular protein determination by FCM, BrefA (1 μM) was added to prevent protein transport.

### Statistical analysis

Significance assessments were performed with GraphPad Prism 9. A two-tailed Student's t-test was used for comparisons in experiments with two conditions. One-way analysis of variance (ANOVA) analysis was used for experiments with more than two groups and corrected by the Bonferroni test for multiple comparisons. All data have been generated from at least three independent biological experiments. P < 0.05 was considered significant.

## Supplementary Material

Supplementary figures and tables.

## Figures and Tables

**Figure 9:**
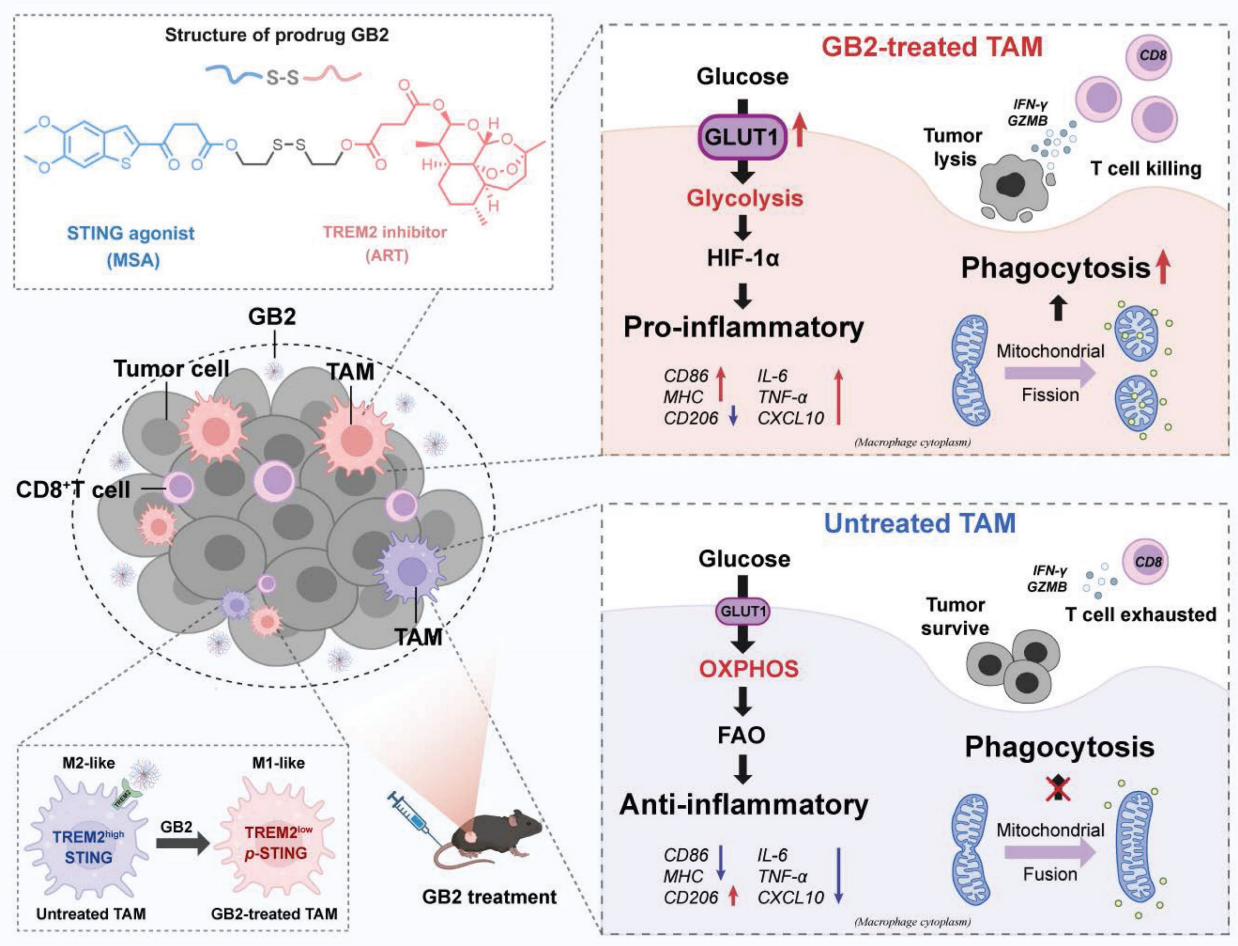
Schematic illustration for prodrug GB2's antitumor mechanism.

## Abbreviations

ART: artesunate
BMDM: bone marrow-derived macrophages
CDN: cyclic dinucleotides
CRC: colorectal cancer
DAP12: DNAX-activation protein 12
FCM: flow cytometry
CLSM: confocal laser scanning microscope
ICB: immune checkpoint blockade
IF: immunofluorescence
IHC: immuno-histochemistry
GEPIA: gene ex pression profiling interactive analysis
GLUT1: glucose transporter 1
GSEA: Gene set enrichment analysis
GSH: glutathione
GZMB: granzyme B
IFN-Ⅰ: type I interferon
IL-6: interleukin-6
iNOS: nitric oxide synthase
ISGs: interferon-stimulated genes
MERC: endoplasmic reticulum-mitochondria contact
NES: normalized enrichment score
NK: natural killer
OS: overall survival
OXPHOS: oxidative phosphorylation
PDI: polydispersity index
RFS: refractory free survival
RNA-seq: RNA-sequencing
ROS: reactive oxygen species
scRNA-seq: single-cell RNA sequencing
STING: stimulator of interferon gene
TAMs: Tumor-associated macrophages
TCGA: the cancer genome atlas
TILs: tumor infiltrating lymphocytes
TEM: transmission electron microscopy
TREM2: triggering receptor expressed on myeloid cells 2
t-SNE: t-distributed stochastic neighbor embedding
