# Supplementary material for: A STING agonist prodrug reprograms tumor-associated macrophage to boost colorectal cancer immunotherapy
Source: Theranostics. 2025 Jan 1;15(1):277–99. doi: 10.7150/thno.101001 (PMC11667223; doi:10.7150/thno.101001)
Supplement: Supplementary file 1 — Supplementary figures and tables. [file thnov15p0277s1.pdf]

Supplementary Information for

A STING agonist prodrug reprograms tumor-associated macrophage to boost colorectal cancer immunotherapy

Aohua Deng[1,2,3#], Renming Fan[1,2#], Yongrui Hai[1,2#], Junyan Zhuang[1,2#], Bingjie Zhang[1,2], Xintong Lu[1,2], Wenhui Wang[1,2], Li Luo[1,4], Ge Bai[1,2], Lei Liang[5✉], Le Yang[4✉], Minggao Zhao[4✉], Gaofei Wei[1,2✉]

# These authors contributed equally to this work.

✉Corresponding authors: Gaofei Wei (weigf0605@163.com), Lei Liang (llknight115@163.com), Le Yang (yanglefmmu@163.com), Minggao Zhao (minggao@fmmu.edu.cn)

This PDF file includes:

Supplementary Figures S1 to S10

Supplementary Table S1 and S2

Supplementary Methods

Chemical synthesis for GB2

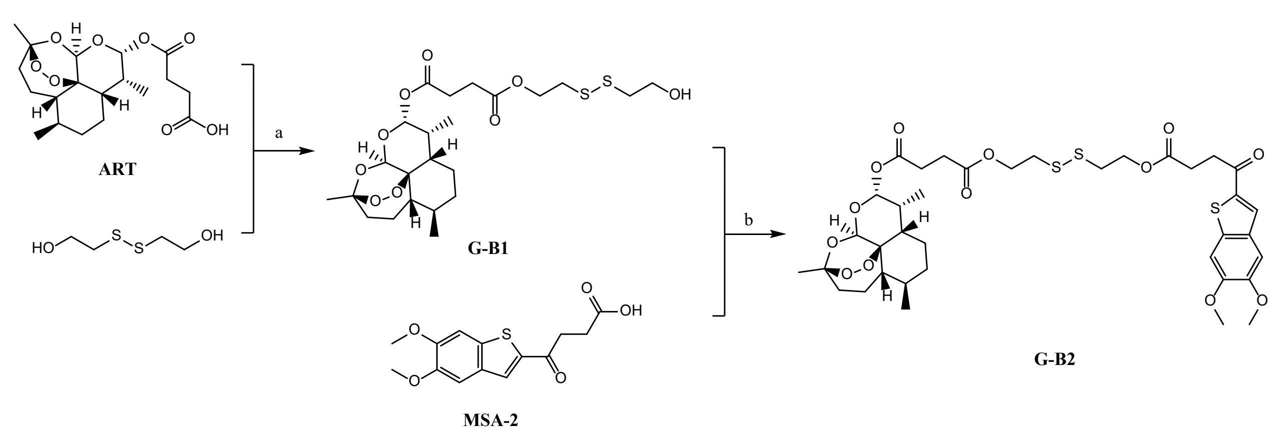

Scheme S1. Synthesis routes for GB2.

Synthesis for GB1 (a): Bis(2-hydroxyethyl) dissulfide (0.12 mmol) was dissolved in 4 ml of dimethyl sulfoxide (DMSO) in a 50-ml round-bottom flask. Then, ART (0.24 mmol) and DMAP (0.48 mmol) were added dropwise sequentially and stirred in an ice bath for 30 minutes. Next, EDCI (0.36 mmol) was added, and the mixture was further stirred at room temperature for 24 hours. The completion of the reaction was monitored by thin-layer chromatography. The target products were purified by column chromatography to give a Transparent oil like substance G-B1 (76 mg, Yield, 61%). ^{1}H NMR (500 MHz, $CDCl_3$): δ 5.78 (d, J = 9.9 Hz, 1H), 5.44 (s, 1H), 4.38 (tt, J = 6.6, 2.0 Hz, 2H), 3.88 (t, J = 6.0 Hz, 2H), 2.94 (t, J = 6.6 Hz, 2H), 2.88 (t, J = 6.0 Hz, 2H), 2.77 – 2.73 (m, 2H), 2.71 – 2.64 (m, 2H), 2.59 – 2.53 (m, 1H), 2.37 (ddd, J = 14.6, 13.4, 4.0 Hz, 2H), 2.03 (ddd, J = 14.5, 4.9, 2.9 Hz, 1H), 1.89 (ddt, J = 13.6, 6.7, 3.6 Hz, 1H), 1.81 – 1.69 (m, 2H), 1.66 – 1.59 (m, 1H), 1.53 – 1.42 (m, 4H), 1.40 – 1.25 (m, 3H), 1.06 – 0.94 (m, 4H), 0.86 (d, J = 7.2 Hz, 3H). ^{13}C NMR (126 MHz, $CDCl_3$): δ 171.98, 171.19, 104.47, 92.26, 91.48, 80.10, 62.62, 60.36, 51.52, 45.19, 41.45, 37.22, 36.93, 36.19, 34.05, 31.76, 29.11, 28.81, 25.91, 24.55, 21.95, 20.20, 12.05.

Synthesis for GB2 (b): MSA-2 was dissolved in 4 ml of dimethyl sulfoxide (DMSO) in a 50-ml round-bottom flask. Then, G-B1 (0.24 mmol) and DMAP (0.48 mmol) were added dropwise sequentially and stirred in an ice bath for 30 minutes. Next, EDCI (0.36 mmol) was added, and the mixture as further stirred at room temperature for 24 hours. The completion of the reaction was monitored by thin-layer chromatography. The target products were purified to give a Transparent oil

like substance G-B2 (133 mg, Yield, 71%). ^{1}H NMR (500 MHz, $CDCl_3$) δ 7.91 – 7.85 (m, 1H), 7.22 (d, *J* = 3.8 Hz, 2H), 5.74 (dt, *J* = 9.9, 1.6 Hz, 1H), 5.36 (d, *J* = 1.4 Hz, 1H), 4.36 – 4.30 (m, 4H), 3.96 – 3.89 (m, 6H), 3.29 (td, *J* = 6.8, 1.4 Hz, 2H), 2.92 – 2.87 (m, 4H), 2.79 – 2.75 (m, 2H), 2.71 – 2.60 (m, 4H), 2.36 – 2.28 (m, 1H), 2.02 – 1.97 (m, 1H), 1.84 (ddd, *J* = 14.1, 6.5, 3.3 Hz, 1H), 1.74 – 1.63 (m, 2H), 1.56 (dt, *J* = 13.7, 4.5 Hz, 1H), 1.43 – 1.36 (m, 4H), 1.28 – 1.18 (m, 3H), 0.97 – 0.89 (m, 4H), 0.83 – 0.78 (m, 3H). ^{13}C NMR (126 MHz, $CDCl_3$) δ 191.78, 172.45, 171.87, 171.05, 151.03, 148.86, 141.04, 136.77, 132.64, 129.16, 105.96, 104.42, 103.58, 92.26, 91.46, 80.09, 62.55, 62.52, 56.20, 56.07, 51.51, 45.19, 37.21, 37.16, 37.05, 36.20, 34.06, 33.57, 31.78, 29.11, 28.79, 28.28, 25.93, 24.55, 21.94, 20.20, 12.05. HRMS (ESI) *m*/*z*: $[M + Na]^+$ calcd for $C_{37}H_{48}O_{13}S_3$, 819.2204; found, 819.2155.

Table S1. Antibodies used for flow cytometry

Antibody	Dilution	Catalog	Manufacturer
CD16/32	1:200	80366	CST
PE Anti-Mouse CD80 (B7-1)	1:40	PE-65076	Proteintech
APC Anti-Mouse CD86 (GL1)	1:100	Apc-65068	Proteintech
FITC anti-mouse CD11c Antibody	1:200	117306	Biolegend
PerCP/Cyanine5.5 anti-mouse CD45 Antibody	1:80	103132	Biolegend
CD3 (17A2) Rat mAb (APC Conjugate)	1:40	24265	CST
CD4 (RM4-5) Rat mAb (FITC Conjugate)	1:200	96127	CST
CD8α (2.43) Rat mAb (PE Conjugate)	1:160	56984	CST
CD11b/ITGAM (M1/70) Rat mAb (PerCP-Cy5.5® Conjugate)	1:80	85601	CST
FITC anti-mouse F4/80 Antibody	1:200	123108	Biolegend
CD86/B7-2 (GL-1) Rat mAb (APC Conjugate)	1:330	84393	CST
Alexa Fluro700 anti-mouse I-A/I-E Antibody	1:200	107622	Biolegend
PE anti-mouse CD206 (MMR) Antibody	1:40	141706	Biolegend
Zombie Violet™ Fixable Viability Kit	1:500	423114	Biolegend

Table S2. Antibodies used for IF, IHC and Western blot

Antibody	Catalog	Manufacturer
TREM2 Polyclonal Antibody	PA5-119690	Invitrogen
Anti-Vinculin	ab129002	Abcam
Anti-GAPDH antibody	ab181602	Abcam
Anti-beta Actin antibody	ab8226	Abcam
Anti-iNOS antibody	ab178945	Abcam
STING (D1V5L) Rabbit mAb	50494	CST
Phospho-STING (Ser365) (D8F4W) Rabbit mAb	72971	CST
Phospho-STING (Ser365) (D1C4T) Rabbit mAb	51865	CST
cGAS (D3O8O) Rabbit mAb	31659	CST
TBK1/NAK (D1B4) Rabbit mAb	3504	CST
Phospho-TBK1/NAK (Ser172) (D52C2) XP® Rabbit mAb	5483	CST
IRF-3 (D83B9) Rabbit mAb	4302	CST
Phospho-IRF-3 (Ser396) (D6O1M) Rabbit mAb	29047	CST
Recombinant Anti-Ki67 antibody (Rabbit mAb)	GB151499-100	Servicebio
Anti-Glucose Transporter GLUT1 antibody	ab115730	Abcam
CD206/MRC1 (E6T5J) XP® Rabbit mAb	24595	CST
Anti-Granzyme B antibody	ab255598	Abcam
Anti-rabbit IgG, HRP-linked Antibody	7074	CST
Goat Anti-mouse IgG H&L	ab6708	Abcam
Goat Anti-Rabbit IgG H&L	ab205718	Abcam

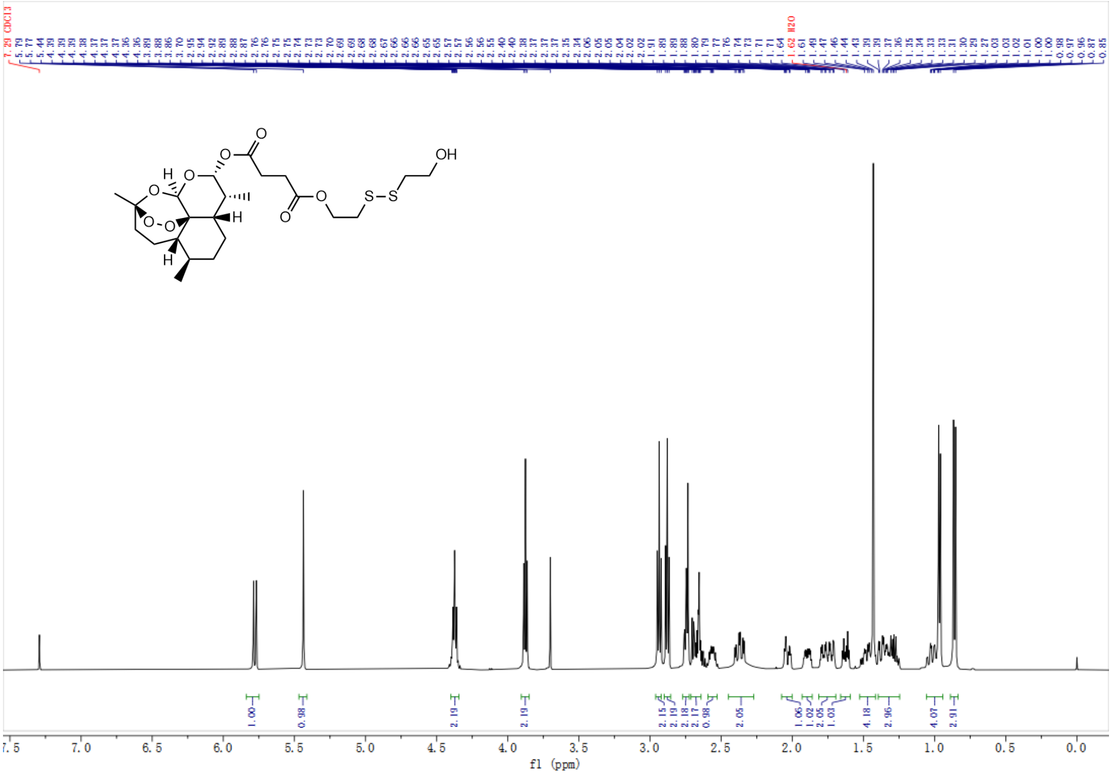

Figure S1. ^{1}H-NMR spectrum of GB1

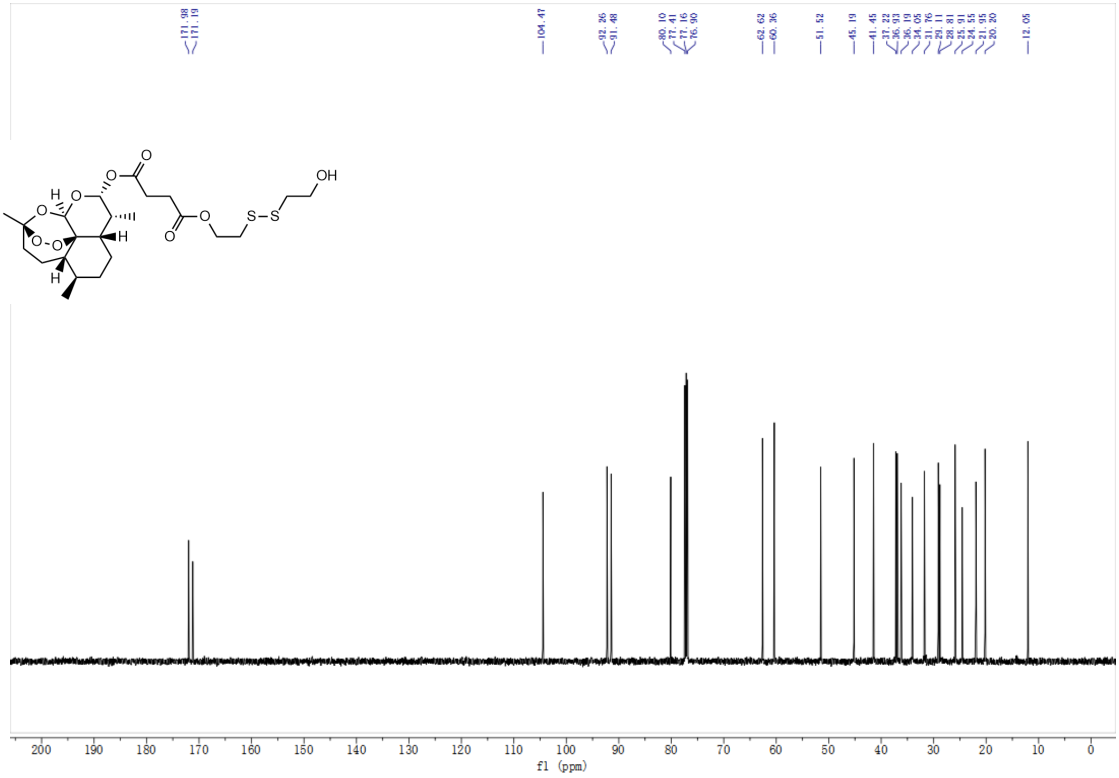

Figure S2. ^{13}C-NMR spectrum of GB1

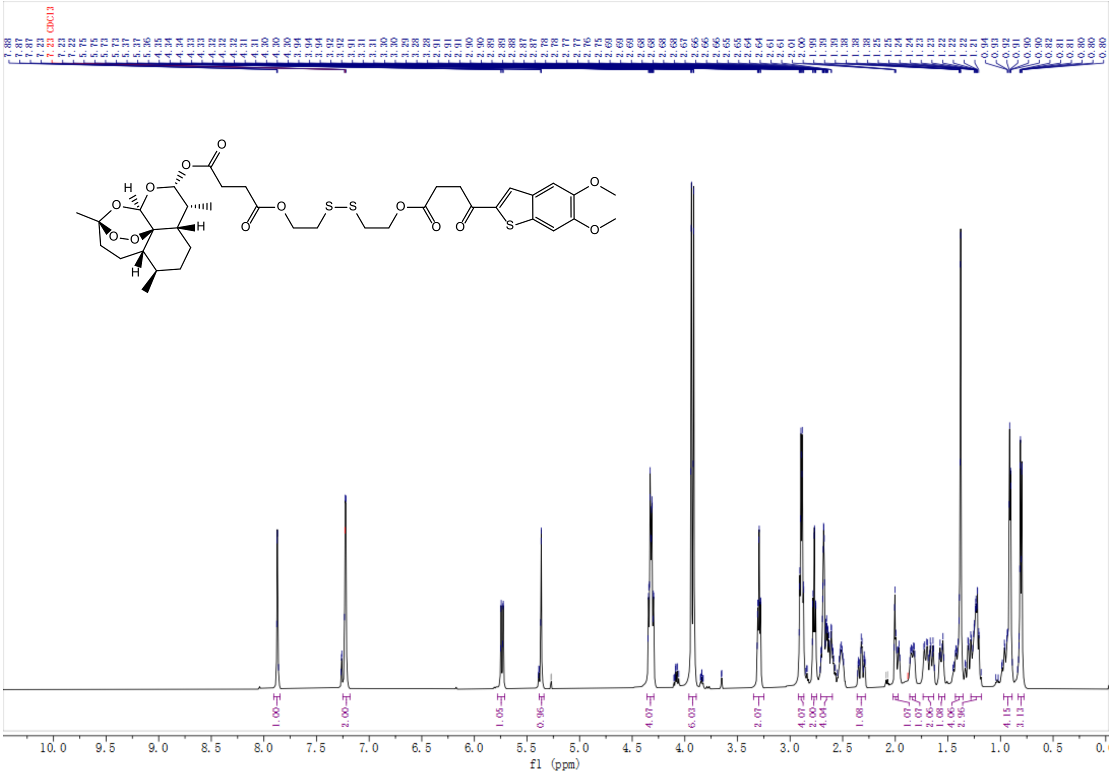

Figure S3. ^{1}H-NMR spectrum of GB2

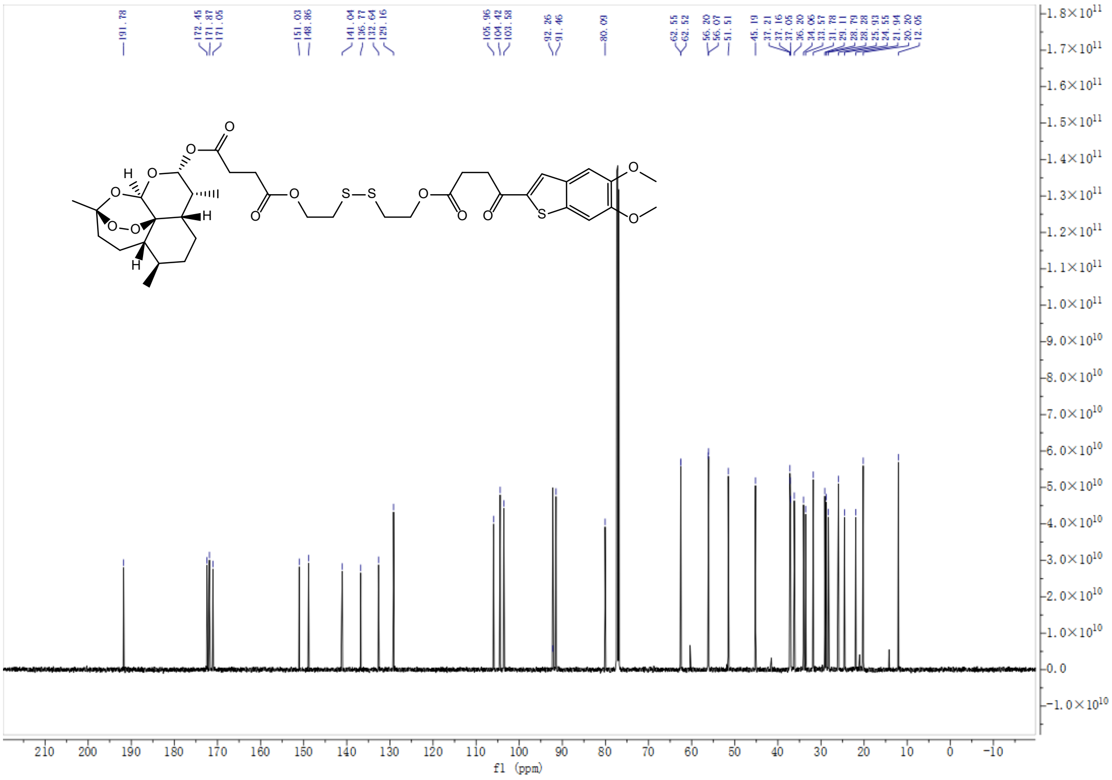

Figure S4. ^{13}C-NMR spectrum of GB2

Monoisotopic Mass, Even Electron Ions
1 formula(e) evaluated with 1 results within limits (up to 50 best isotopic matches for each mass)
Elements Used:
C: 37-37 H: 48-48 O: 13-13 Na: 0-1 S: 3-3
G-B2 14 (0.145)
1: TOF MS ES+

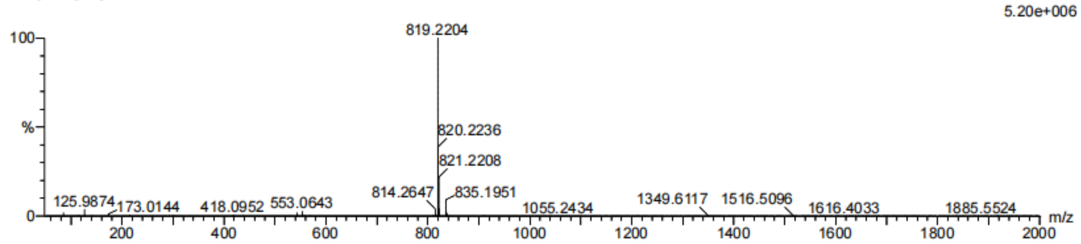

Minimum:				-1.5					
Maximum:		5.0	10.0	50.0					
Mass	Calc. Mass	mDa	PPM	DBE	i-FIT	Norm	Conf(%)	Formula	
819.2204	819.2155	4.9	6.0	13.5	341.4	n/a	n/a	C37 H48 O13 Na S3	

Figure S5. HR-MS spectrum of GB2

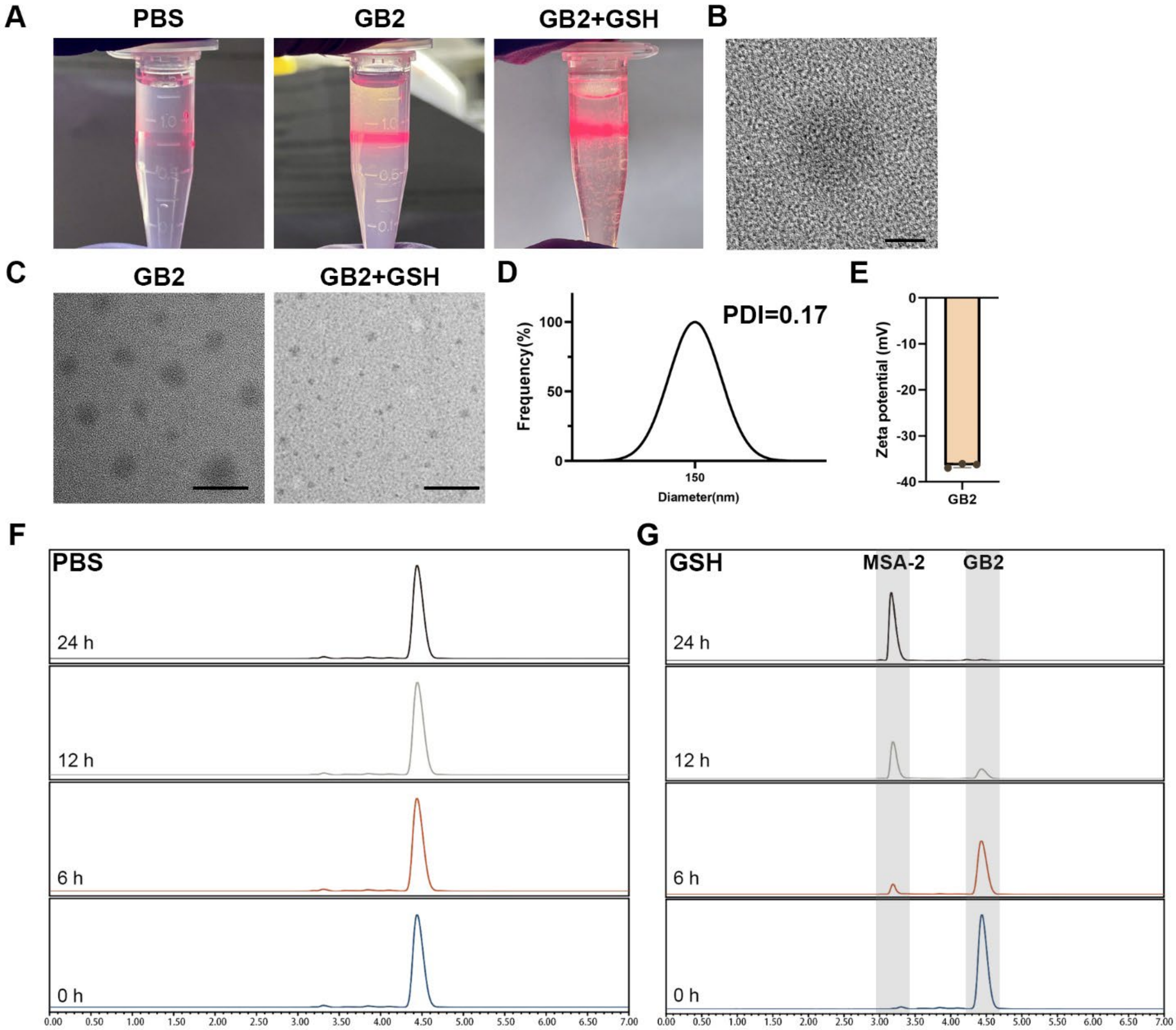

Figure S6. Evaluation on self-assemble property of GB2. (A) Tyndall effect of GB2. (B) TEM image for GB2. Scale bar: 50 nm. (C) TEM image of GB2 and its GSH-responsive reaction. (D) Diameter

distribution and PDI of GB2 particles. Scale bar: 200 nm. (E) The surface charge of GB2. (F) The stability of GB2 in PBS for 24 h. (G) The GSH-responsive reaction of GB2 for 24 h.

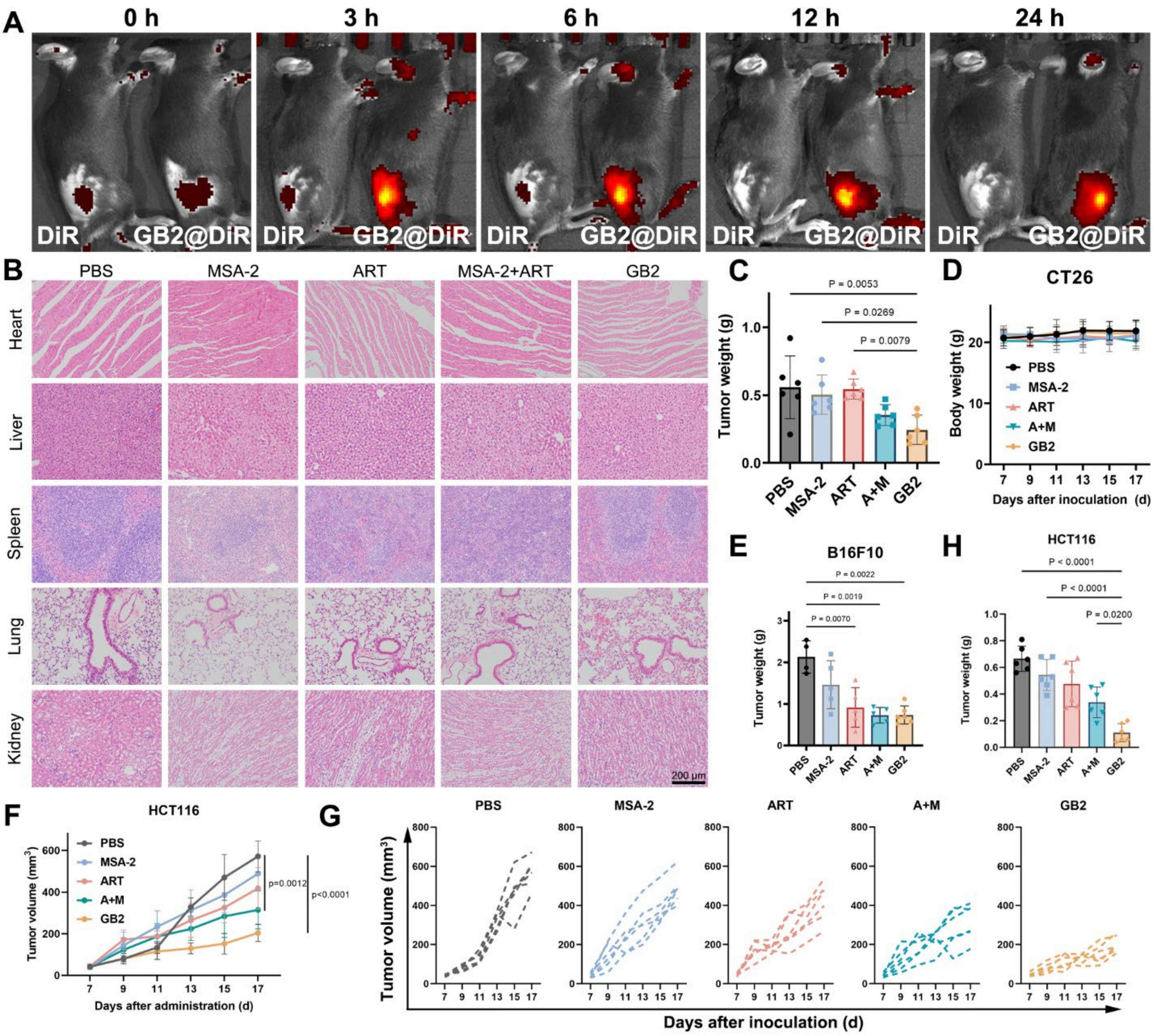

Figure S7. Pharmacological analysis for GB2 in mouse tumor models. (A) In vivo distribution of GB2@DiR determined by fluorescence bio-imaging. (B) HE staining images of major organs in the MC38 model group. Scale bar: 200 μm. (C) Weight of CT26 tumors at day 17 (n = 6). (D) Mice weight of CT26 model records (n = 6). (E) Weight of B16F10 tumors at day 17 (n = 6). (F) Growth curve for HCT116 tumors with indicated treatment (n = 6). (G) Growth of individual HCT116 tumors (n = 6). (H) Weight of HCT116 tumors at day 17 (n = 6).

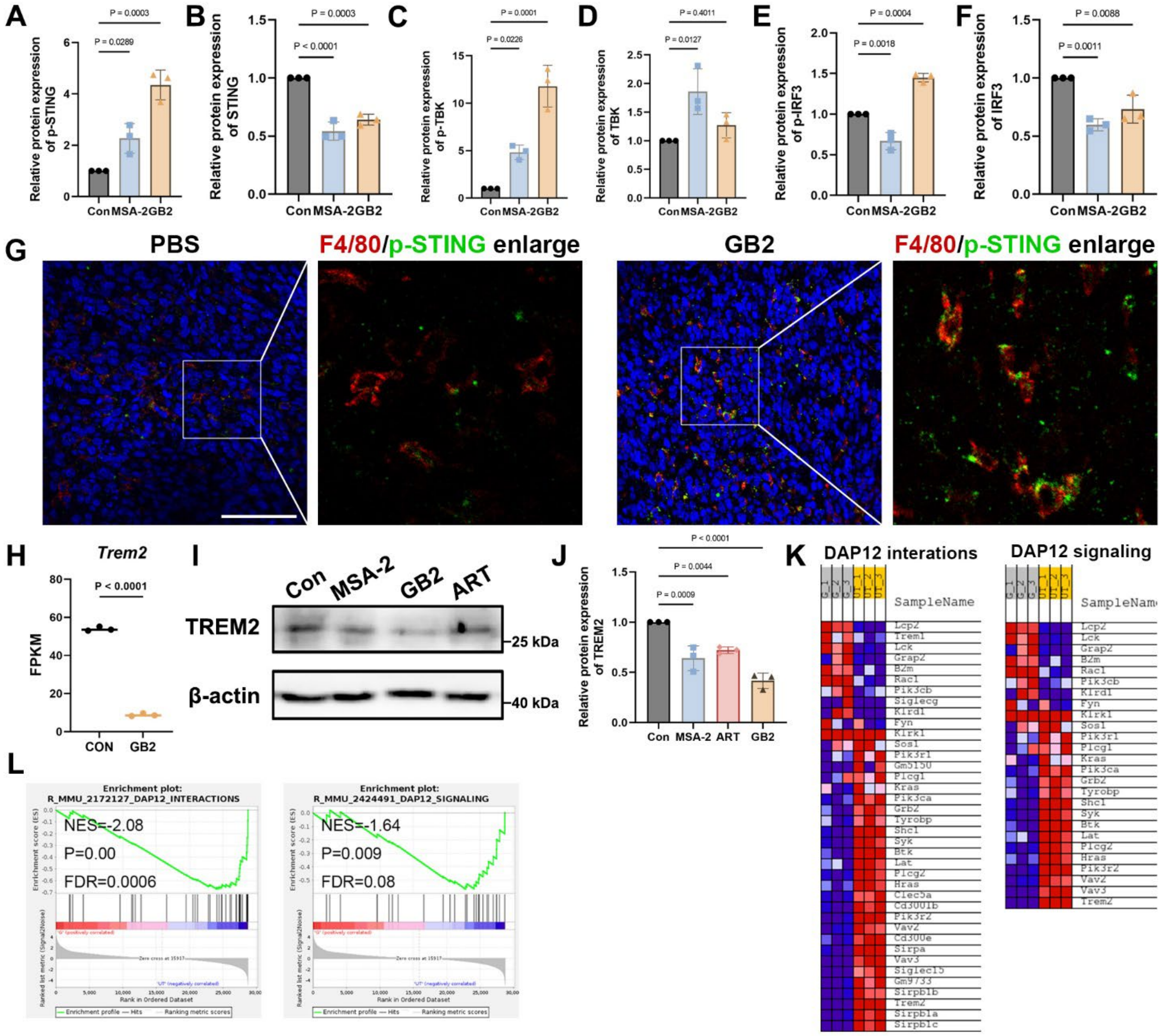

Figure S8. GB2 inhibits TREM2, activates p-STING, and enhances phagocytosis capacity in RAW264.7 and BMDM. (A-F) Relative protein quantification of STING-related proteins. (G) The CLSM images of macrophage p-STING activation *in vivo*. Scale bar: 90 µm. (H) GB2 downregulates Trem2 gene expression in RAW264.7 cells. (I) GB2 downregulation of TREM2 expression in BV-2 microglial cells. (J) Relative protein quantification of TREM2 (K-L) GB2 downregulates genes related with DAP12 signaling pathways in RAW264.7 cells.

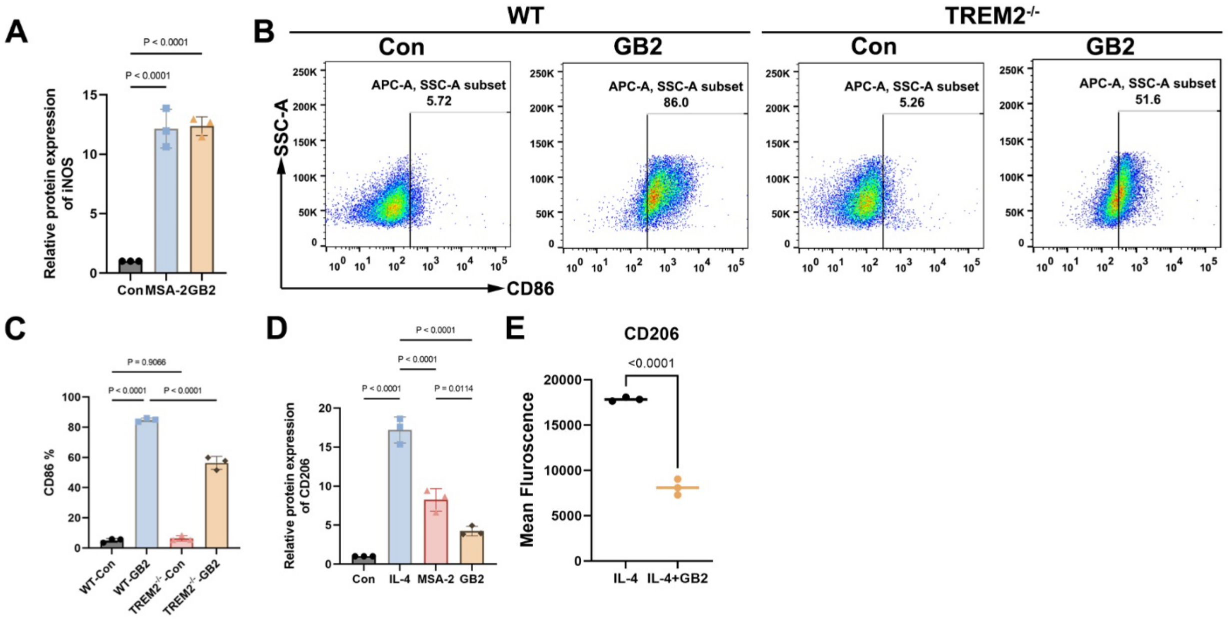

Figure S9. GB2 promotes M1-like macropahge phenotype by upregulating antigen presentation and initiating inflammatory response, and reverses IL4-induced M2-like macrophage phenotype. (A) Relative protein quantification of iNOS proteins. (B-C) FCM graphs and statistic analysis for RAW264.7 (D) Relative protein quantification of CD206. (E) Quantitative analysis was conducted by calculating mean fluorescence in indicated groups (n = 3).

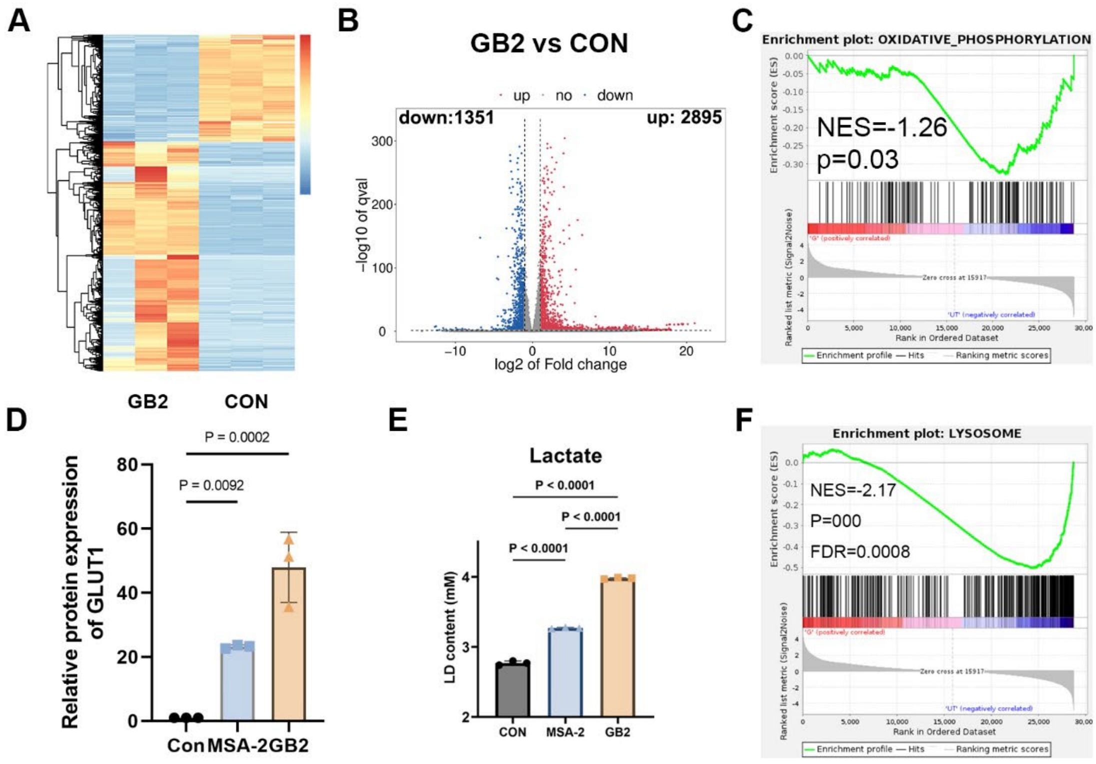

Figure S10. Mechanism analysis for GB2-treated macrophages. (A-B) Heatmap and volcano plot for

differentially expressed genes in RAW264.7 cells. (C) GB2 downregulates genes in OXPHOS pathway. (D) Relative protein quantification of GLUT1. (E) Lactate content in treated or untreated RAW264.7 cells. (F) GSEA plot for differentially expressed lysosome pathway in RAW264.7 cells (GB2 vs CON).
